# Rapid Chiral Analysis of Drugs with Multiple Stereocenters via High-Resolution Ion Mobility

**DOI:** 10.1021/acs.analchem.6c03422

**Published:** 2026-07-29

**Authors:** Benjamin K. Blakley, Jody C. May, Valeria Guidolin, Bao Nguyen, John A. McLean

**Affiliations:** † Department of Chemistry, Center for Innovative Technology, Vanderbilt Institute of Chemical Biology, Vanderbilt-Ingram Cancer Center, and Vanderbilt Institute for Integrated Biosystems Research and Education, 5718 Vanderbilt University, Nashville, Tennessee 37235-1822, United States; ‡ Pfizer, Inc., Pharmaceutical Sciences Small Molecules (PSSM), Groton, Connecticut 06340, United States

## Abstract

The increasing structural complexity of small-molecule therapeutics requires sensitive analytical techniques that facilitate rapid chiral analysis of drugs with multiple chiral centers. Here, we demonstrate ultrafast chiral separations for compounds possessing 3 chiral centers (8 stereoisomers, 4 enantiomer pairs) using gas-phase ion mobility spectrometry–mass spectrometry (IM-MS). Separations are achieved using spontaneous noncovalent copper–tyrosine complexation.This complexation strategy converts all stereoisomers into diastereomers that are structurally distinguishable by IM-MS. Notably, this strategy is over 3 orders of magnitude faster than traditional liquid-phase chiral chromatography (ms vs min, respectively). Using IM-MS, we achieved direct baseline separation of enantiomer pairs and progress toward differentiation of all 8 stereoisomers. Results suggest that higher-order complexes (i.e., copper–tyrosine-bound drug dimers) enhance chiral differentiation at the expense of increased spectral complexity. Exchanging the chirality of the amino acid for complexation resulted in an inversion of separation elution ordering. Peak fitting analysis suggests that an IM resolving power of several thousand is necessary to resolve all stereoisomers for quantitative purposes, which is approximately an order of magnitude higher than the current state-of-the-art. Collectively, these findings demonstrate the speed and versatility of gas-phase ion mobility spectrometry for complex chiral analysis.

## Graphical abstract

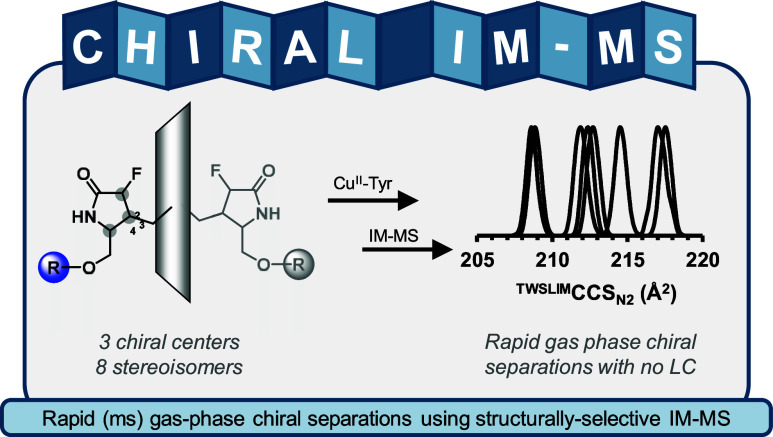

## Introduction

Effective drug discovery, development, and processing necessitates rapid analytical methodologies to differentiate all stereoisomers of a given active pharmaceutical ingredient (API). However, the increasing prevalence of drugs with multiple chiral centers presents a growing analytical challenge. A search of the NCATS Inxight Drugs database returns a total of 269 prescription drugs approved by the U.S. Food and Drug Administration in the past decade. Of these drugs, 250 (93%) are small molecules of less than 1000 Da and 158 are chiral compounds (Figure 1A).^1^ While 37% of these chiral compounds possess a single chiral center, the majority (63%) have multiple chiral centers, and over a third (38%) contain 3 or more chiral centers (Figure 1B). This increased stereochemical complexity places a considerable burden on existing chiral analysis techniques, which are often both resource- and time-intensive to implement. Such analytical constraints contribute to the high costs associated with pharmaceutical research and development, a key factor in the monetary costs to bring a single drug to market—estimated to be $2.87 billion USD in 2016 ^2^ and $4.46 billion USD as of 2024.^3^ Consequently, there is substantial demand within the pharmaceutical industry for analytical techniques that facilitate faster and more cost-effective chiral analysis.

**Figure 1:**
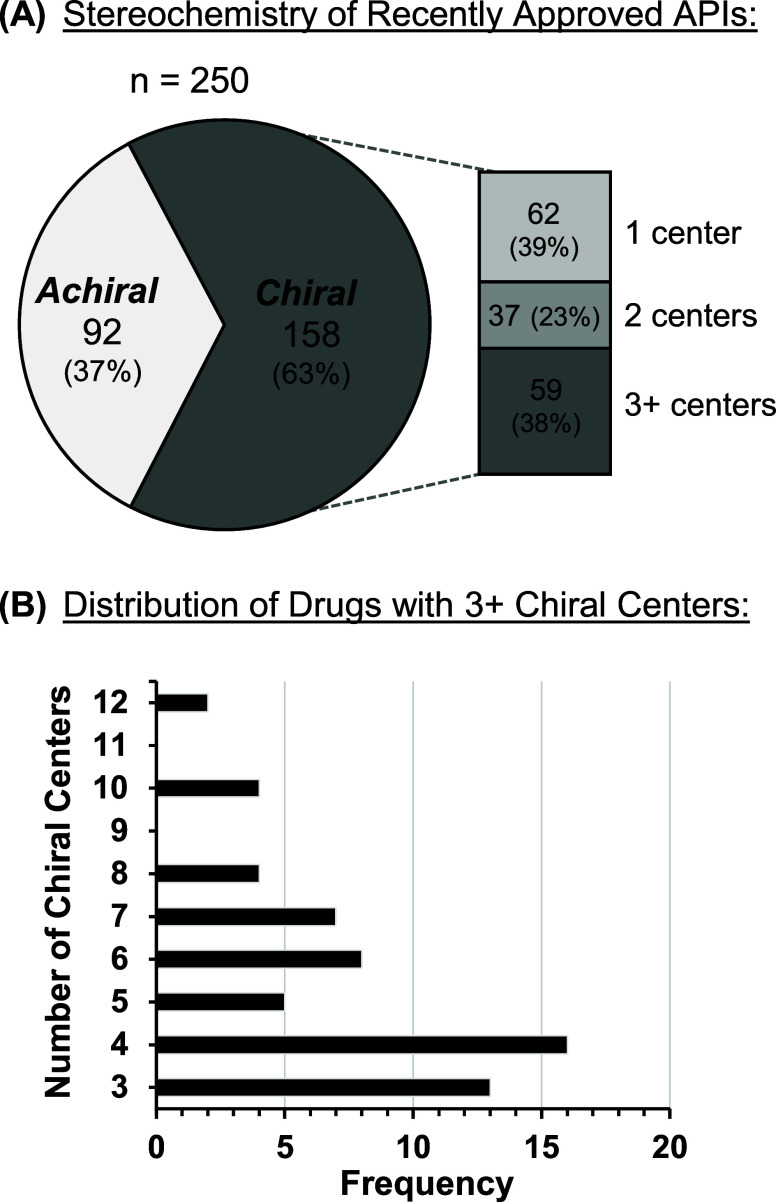
Of the 269 prescription drugs approved by the U.S. Food and Drug Administration in the past decade, 250 have a molecular weight less than 1000 Da. (A) Breakdown of achiral and chiral drugs, as well as number of chiral drugs with 1, 2, and 3+ chiral centers. (B) Frequencies for the number of chiral centers shown for drugs with 3 or more chiral centers. Search of the NCATS Inxight Drugs database (accessed August 25, 2025).

Existing mass spectrometry (MS)-based methods for differentiating enantiomers, such as energy-resolved measurements via the kinetic method^4^ and ion trap MS with symmetry breaking,^5^ are typically restricted to resolving simple enantiomeric pairs with a single chiral center, which significantly limits their utility for the analysis of more complex pharmaceutically relevant compounds containing multiple chiral centers. Kinetic method studies involving multiple analytes would be hindered by multiple MS/MS branching ratios and would require further investigations into competitive effects arising from the additional analyte forms that undergo fragmentation. Furthermore, while symmetry-breaking approaches using an ion trap do not require the introduction of analytes to a chiral environment, this technique will require extensive reproducibility studies before widespread adoption in pharmaceutical pipelines.

While spectroscopic techniques such as nuclear magnetic resonance (NMR)^6^ and circular dichroism (CD)^7^ offer rapid chiral analysis, their utility is often limited by low sensitivity and lack of specificity for multichiral center compounds. For chiroptical techniques, such as CD, the molecule must carry a chromophore that absorbs in the spectral detection range. For conventional NMR, enantiomers are isochronous and require symmetry breaking by introducing a chiral modifier either through covalent derivatization or through the formation of transient diastereomers by means of a chiral solvating agent. To overcome some of these limitations, state-of-the-art chiral separations often incorporate multidimensional separation strategies, which typically involve an initial RPLC step to separate diastereomers, followed by a second dimension of analysis for pairwise enantiomer differentiation. This second stage of analysis can be achieved using either spectroscopic or spectrometric approaches or an additional chiral LC dimension (2D-LC).^8^ However, these chiral LC methods are often isocratic and analyte-specific, requiring significant time and resources for method development and empirical screening. This analytical bottleneck has created a substantial demand within the pharmaceutical industry for more efficient and rapid methodologies to address the complexities of multichiral center drug characterization.

Ion mobility-mass spectrometry (IM-MS) enables rapid (ms) gas-phase separation of ions on the basis of their collision cross-section (CCS) and mass-to-charge (*m*/*z*). This dual-parameter separation provides a powerful analytical tool for the sensitive and rapid differentiation of isomers based on their distinct molecular structure.^9,10^ Recent advancements in high-resolution ion mobility spectrometry (HRIM) instrumentation, such as trapped ion mobility spectrometry (TIMS), differential mobility spectrometry (DMS/FAIMS), cyclic ion mobility spectrometry (cIMS), and traveling wave structures for lossless ion manipulation (TWSLIM), have significantly enhanced isomeric characterization across a broad range of CCS and molecular mass. HRIM techniques operate with a high separation efficiency, with resolving powers (R_P_) greater than 250, and can be further integrated with solution-phase noncovalent complexation strategies, for example, copper–amino acid complexation, to facilitate separation of enantiomers bound within a structurally distinct diastereomeric complex.^11–16^ In a 2016 study from Nagy and Clemmer, manganese–amino acid and protonated amino acid complexes were used to resolve a suite of 16 glucose isomers using multidimensional ion mobility.^17^ The stereoselectivity of these complexes is governed by the Pirkle Rule, which states that in order to be enantioselective, there must be three simultaneous interactions between the analyte and selector, with at least one interaction dependent on the analyte’s stereochemistry. Additional divalent metals such as nickel, magnesium, and zinc^16^ have been explored for ligand exchange, while studies investigating simpler complexation modalities have used monovalent metals such as sodium,^18^ silver, lithium,^19^ potassium,^20^ and barium.^21^ We emphasize that copper was chosen for this study due to its d^9^ electron configuration facilitating facile complexation via Jahn–Teller distortions, and copper also exhibits high affinity for amino acids and a unique and diagnostic isotope distribution, which collectively serve to increase the likelihood that the complexes formed are enantioselective, tightly bound, and easily identifiable. However, prior studies reported superior performance from metals other than copper in specific applications. Other isomer-selective complexation modalities have also been combined with ion mobility analysis to achieve chiral recognition, including self-association complexes,^18,22^ covalently labeled analytes,^23,24^ and cyclodextrin host/guest inclusion complexes.^25–27^ Other emerging complexation approaches include additional macrocycles such as crown ethers^28,29^ and cucurbiturils.^30^

In this report, we describe a workflow for the HRIM-MS analysis of multichiral center APIs following complexation to a copper-tyrosine core and demonstrate analytical separations of multichiral center compounds on a time scale that is approximately 3 orders of magnitude faster than traditional chiral LC–MS. Previous work suggests that aromatic amino acids are particularly effective as chiral selectors,^11,12^ presumably due to the aromatic ring as a point of interaction with copper. Tyrosine was selected as a reference ligand for this study because of its aromaticity and the additional inductive effects introduced by the hydroxyl in its phenol group, which we hypothesize enhances stereoselectivity. This rapid chiral separation capability enabled the stereochemical characterization of two drug scaffolds containing 3 chiral centers: (1) an IRAK4 inhibitor, Zimlovisertib; and (2) Zimlovisertib’s γ-lactam scaffold (Figures 2A,S1 and Table S1). While prior work has demonstrated the effectiveness of the chiral selectors used here, the novel aspects of this current study are in applying these strategies to a complex chiral compound, namely, one with three chiral centers bound within a single ring. Importantly, all of the possible stereoisomers of these compounds were synthesized and validated as part of a major pharmaceutical drug discovery effort,^31^ providing a rare opportunity to evaluate and validate chiral recognition strategies via ion mobility on a complete isomer system. NMR characterization of synthesized compounds for each of the drugs’ eight pure isomer forms can be found in Figures S2–S17.

**Figure 2:**
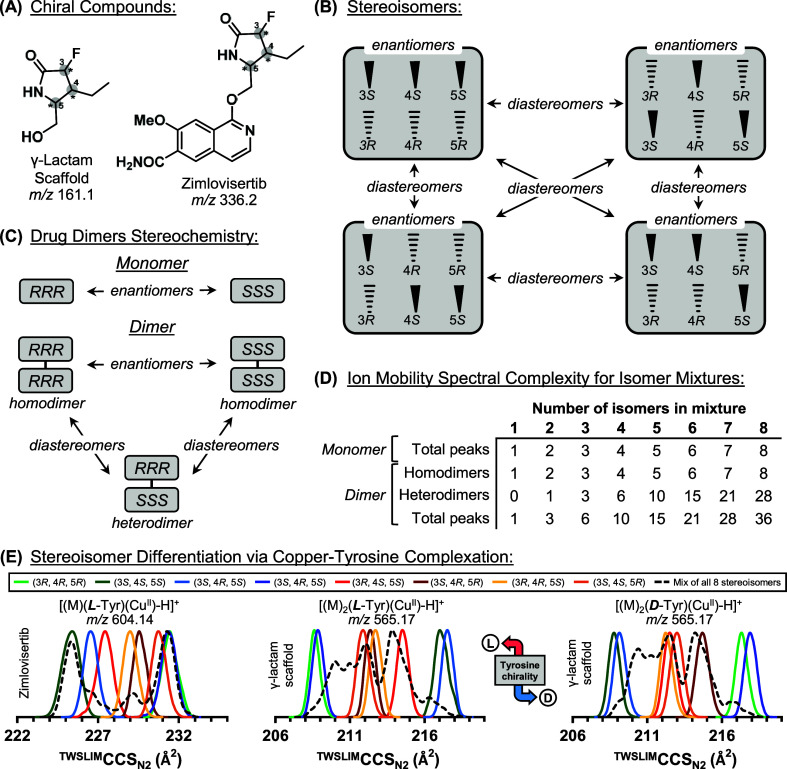
(A) Structures and (B) stereoisomers for chiral substrates investigated. (C) A visualization of the increased number of possible complexes afforded by a drug dimer, leading to heightened degrees of spectral complexity for noncovalent complexes. (D) The number of possible peaks for monomeric and dimeric complexes as a function of the number of components present in a mixture. (E) HRIM differentiation of stereoisomers from mixtures of 8 total isomers.

## Experimental Section

### Chemicals

Copper­(II) acetate and *l* -tyrosine were obtained from Sigma-Aldrich, and Optima LC–MS grade methanol and water were obtained from Fisher Scientific. Enantiopure compounds for each stereoisomer were obtained from Pfizer collaborators. Synthetic details for the γ-lactam and full drug have been described previously.^31^ Stereoisomers were prepared as part of a fragment-based drug discovery process that sought to optimize a target for the ligand and lipophilic intensity, as well as enzyme and cellular potency. For each compound, ^1^H NMR spectra were recorded in DMSO–D_6_ at 500 MHz using Bruker spectrometers. All chemicals were used as received. Table S1 is a full summary of reagents and vendor sources, as well as the stereochemistry and PF number for stereoisomers under evaluation. Corresponding structures can be found in Figure S1. ^1^H NMR spectra for each analyte can be found in Figures S2–S17.

### Sample Preparation

1 mg/mL stock solutions of each compound were prepared using a 1:1 methanol/water solution. Stock solutions were then sonicated for 15 min and decanted to remove any insoluble residue. Samples for IM-MS analysis were prepared by combining the drug, copper­(II) acetate, and *l* -tyrosine to a final equimolar concentration of 20 μM using 1:1 methanol/water.

### Instrument Parameters

Samples were directly infused (10 μL/min) into an ESI source (Jet Stream, Agilent Technologies, Santa Clara, CA) into one of two IM-MS instruments operating in the positive ion mode with the QTOF tuned to a low mass range. Preliminary ion mobility measurements were obtained using a drift tube IM-MS (DTIMS, 6560 IM-QTOF, Agilent). High-resolution ion mobility measurements were performed using a SLIM-based traveling wave HRIM-MS (TWSLIM-MS, MOBIE, MOBILion Systems, Chadds Ford, PA) interfaced with a quadrupole-time-of-flight mass spectrometer (6546, Agilent). Schematics for both instruments can be found in Figure S18. Instrument parameters were previously optimized for the transfer and detection of noncovalent complexes, as described by Zlibut et al. for DTIMS^11^ and by Blakley et al. for TWSLIM.^12^

### Data Processing and Software

PNNL PreProcessor (3.0) was used for CCS calibration of the entire profile data from arrival time to CCS.^32^ Drift tube arrival time measurements were converted to ^DT^CCS_N2_ values by using the single-field linear equation CCS calibration method.^33^ TWSLIM arrival time measurements were converted to ^TWSLIM^CCS_N2_ via a third-order polynomial with no additional correction applied to align data to ^DT^CCS_N2_. A homologous series of symmetrically branched hexakis­(fluoroalkoxy) phosphazenes within a commercial tuning mixture (ESI-L, Agilent) were used as calibrants. The CCS-calibrated IM profile data were manually extracted from Agilent MassHunter IM-MS Browser (version 10.0.1.10039) and imported into Microsoft Excel for further peak analysis. Peak centroids were extracted from MassHunter and used to determine the experimental CCS values of the individual peaks. Details of the fitting procedure and peak metrics used to assess separation efficiency can be found in the Supporting Information.

## Results

For drugs with 3 chiral centers, there are 8 total stereoisomers containing 4 pairs of enantiomers (Figure 2B). This imposes inherent spectral complexity on IM data, which can be further exacerbated by the incorporation of drug multimers into ternary metal–amino acid complexes. As recently noted by Cooper-Shepherd et al., for dimers of chiral drugs, both homodimers (e.g., *R*,*R* or *S*,*S*) and heterodimers (e.g., *R*,*S* or *S*,*R*) can form.^18^ This introduces additional spectral features that require elucidation using HRIM (Figure 2C). The number of heterodimers scales with the total number of isomers in the mixture, where the mathematical enumeration of the combined number of homodimers and heterodimers follows a triangular sequence. Thus, a dimeric complex measured in a mixture of 8 stereoisomers will have a total of 36 features (Figure 2D). Figure 2E contains overlaid HRIM profiles for the 8 individual isomers, as well as HRIM distributions for a mixture of all 8 stereoisomers, using the same copper–tyrosine complexation strategy for both multichiral systems analyzed in this study. Tyrosine was selected over other amino acid chiral reference ligands, such as histidine, due to initial DTIMS observations showing both a higher number of target complexes forming and a higher degree of enantioselectivity, as evaluated using the γ-lactam scaffold. Table S2B contains ^DT^CCS_N2_ measurements for γ-lactam copper–histidine complexes. While we did not explicitly conduct a limit of detection (LOD) study, previous work indicates that the LOD for IM-MS is conservatively in the picomolar range for relatively stable analytes (i.e., peptides, vitamin D).^34,35^ However, for ternary copper complexes that are more labile, we cannot detect features with satisfactory peak shapes below a concentration of approximately 1 μM, though specific complexes that form more favorably can be detected at considerably lower concentrations. This sensitivity is greater than that of NMR, which requires highly pure and high-concentration analytes (high μM to mM) for detection.^36,37^ However, the direct infusion work presented here is less sensitive than LC–MS, largely due to ion suppression from competing ionization, though we note that all complexes studied here contain a charge carrier, copper­(II), so their ion yields should still be high.

For the Zimlovisertib-incorporated [(M)­( *l* -Tyr)­(Cu^II^)–H]^+^ complex, percent differences in CCS between adjacent peaks ranged between 0.08% and 0.72% (Table S2A), enabling the differentiation of 4/8 (50%) peaks at an experimental R_P_ of approximately 214. Characterization of the γ-lactam was considerably more challenging despite higher percent differences in CCS between individually measured isomers (ΔCCS_%_ between 0.12% and 1.43%). Difficulty in resolving the γ-lactam isomers is a direct consequence of the spectral complexity imposed by the presence of heterodimer peaks. Here, we achieved differentiation of 7/36 peaks (19%) from a mixture of all 8 stereoisomers. Complexes presented in the main text of this study ([(M)­( *l* -Tyr)­(Cu^II^)–H]^+^ and [(M)_2_( *l* -Tyr)­(Cu^II^)–H]^+^ for Zimlovisertib and the γ-lactam scaffold, respectively) were selected based on (1) high ion intensities sufficient for analysis and (2) differences in CCS observed between isomerically pure complexes, in both DTIMS (Figures S19–S21 and Table S2) and TWSLIM data (Figures S22 and S23).

For each drug analyte, the extent of differentiation for the four corresponding enantiomer pairs was evaluated. For the full drug, chiral differentiation was achieved for all 4 enantiomer pairs, with two (blue and green) exhibiting baseline separation (R_P‑P_ > 2.0), and two (red and yellow) being resolved at greater than half height (R_P‑P_ of 1.24 and 0.96, respectively) (Figure 3A). Similar results were observed for the smaller γ-lactam scaffold (Figure 3B), where in two cases (blue and green), both the homodimer and the heterodimer complexes are completely resolved. In these cases, the heterodimer is a useful diagnostic for identifying dimeric complexes in HRIM data. However, for the less-resolved enantiomer pairs (red and yellow), the presence of the heterodimer complex interfered with the resolution of the homodimer complexes—resulting in lower separation than would be expected based on the peak spacing of enantiopure complexes. Preliminary quantitation experiments are shown in Figure S24, where analysis of the copper–tyrosine dimer in a mixture of enantiomers (3*R*, 4*S*, 5*R*) and (3*S*, 4*R*, 5*S*) at various ratios (10:1, 5:1, 2:1, 1:1, 1:2, 1:5, and 1:10) enabled quantitation of enantiomeric excess using a protocol for drug dimers published by Lee and co-workers in 2024.^22^ Furthermore, MS/MS of the [(M)_2_( *l* -Tyr)­(Cu^II^)–H]^+^ lactam complex yields two characteristic dissociation pathways: loss of the analyte ligand and loss of the tyrosine reference ligand. A representative CID spectrum is found in Figure S25. Based on the literature precedent for the kinetic method, we believe that these complexes differ in their stabilities, suggesting that MS/MS breakdown curves could be used to resolve stereoisomers here. However, this work is beyond the scope of the current study. We tentatively attribute differences in intensities for complexes in mixtures to differences in gas-phase stability, further underscoring the potential of MS/MS to provide additional analytical insights into the formation and stability of these stereoisomer-incorporated complexes.

**Figure 3:**
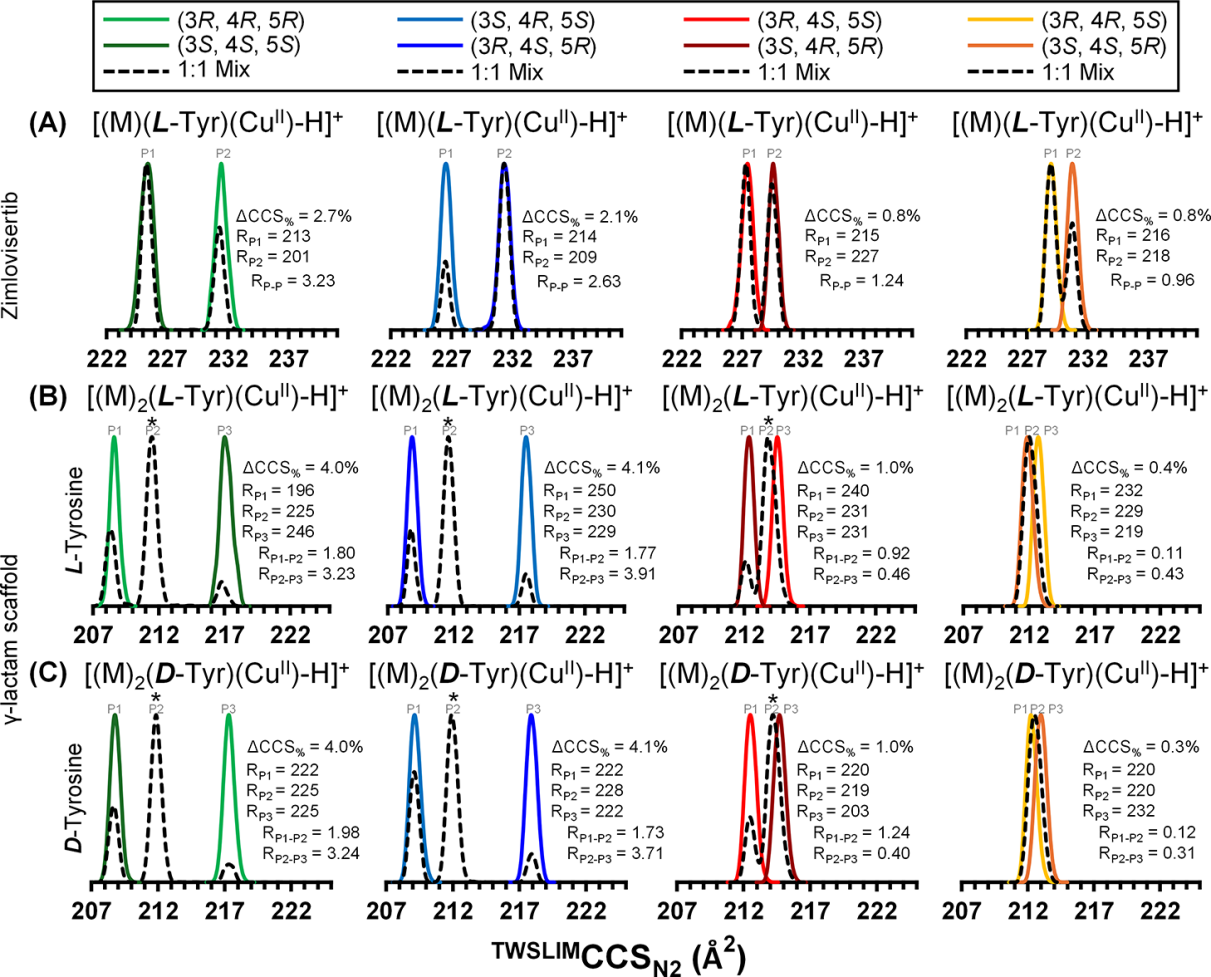
Differentiation of enantiomer pairs using noncovalent complexation for (A) the full drug incorporated into a [(M)­( *l* -Tyr)­(Cu^II^)–H]^+^ complex, (B) the γ-lactam scaffold in a [(M)_2_( *l* -Tyr)­(Cu^II^)–H]^+^ complex, and (C) the γ-lactam scaffold in a [(M)_2_( *d* -Tyr)­(Cu^II^)–H]^+^ complex. The lower two panel shows the effects of swapping the chirality of the selector. Notably, chirality inversion of the selector results in arrival order inversion of separation complexes but preserves the IM profiles for the 1:1 mixture of enantiomers. For reference, two peaks are 10% separated at an R_P‑P_ of 0.61, 50% separated at R_P‑P_ 0.83, 90% separated at R_P‑P_ 1.23, and baseline (100%) separated at R_P‑P_ 2.0 or greater.

These observations underscore the complicated interplay of trade-offs arising from utilizing a metal–amino acid drug dimer for multichiral center separations. We speculate that combining two chiral selection mechanisms (here, self-association from the drug dimer and ligand exchange from the metal–amino acid cluster) can have synergistic effects, imparting pronounced structural modifications and thus higher measurable differences in CCS on isomerically pure complexes compared to results achieved using either approach individually. However, this multimodal strategy also introduces considerable spectral complexity to mixture data due to mixed chirality complexation, imparting a counterintuitive challenge on the separation of multicomponent mixtures.

We also investigated inverting stereochemistry of the amino acid chiral reference ligand on multichiral center systems. Previous reports for analytes with one chiral center demonstrate that inverting the stereochemistry of the chiral reference ligand results in arrival order inversion of the stereoselective complexes. Notably, the IM profiles of the corresponding racemic mixtures were reproduced regardless of the reference ligand chirality, suggesting a highly conserved gas-phase structure is adopted in these noncovalent complexes. This study was complemented by computational modeling, which suggested that the stereoselectivity of these noncovalent complexes is enhanced when the gas-phase structure of the copper-reference ligand core is conserved.^11,12^ As shown in Figure 2E, inverting tyrosine chirality from *l* to *d* results in arrival order inversion between enantiomer pairs. Additionally, HRIM profiles for mixtures of all 8 stereoisomers are extremely similar, but inverted, for complexes incorporating either *l* - or *d* -tyrosine, suggesting that previous observations are generalizable to multichiral systems. As expected, these observations were consistent for enantiomer pair separations. When tyrosine’s chirality is inverted from *l* to *d* , nearly identical HRIM profiles and degree of separation are observed between enantiomer pairs despite an inversion in arrival order (Figure 3C). Moreover, heterodimer peaks retain the same CCS even when the chirality of tyrosine is switched. This data underscores our previous observations and emphasizes the complexity of chiral separations.

To better understand the spectral complexity introduced by the formation of these heterodimer complexes, we sought to experimentally deconvolute the copper–tyrosine dimer mixture of 8 stereoisomers. Samples containing each possible binary mixture of γ-lactam diastereomers (24 total) were mixed with copper–tyrosine and analyzed via HRIM (Figure S26) to elucidate each of the 24 heterodimers in the [(M)_2_( *l* -Tyr)­(Cu^II^)–H]^+^ distribution of the γ-lactam. Gaussian fits were then used to describe TWSLIM distributions such that a mean (representing the ^TWSLIM^CCS_N2_ of a given complex) and standard deviation (representing FHWM, the peak full width at half the maximum height) were obtained for both homodimers and heterodimers. This peak fitting procedure was repeated on the enantiopure data for the Zimlovisertib [(M)­( *l* -Tyr)­(Cu^II^)–H]^+^ distribution. This information was used to reproduce HRIM distributions for mixtures of all 8 stereoisomers by first normalizing Gaussian fits to experimentally observed intensities. Peak intensities were then incrementally adjusted to minimize residuals (Figure S27). (Statistical data obtained from peak fitting is shown in Table S4.) Figure 4 shows a comparison between predicted and experimental HRIM distributions for mixtures of all 8 stereoisomers—our predicted spectra differ from experimentally measured intensities by less than 10% and show close alignment to experimental data, as evidenced by minimal residuals.

**Figure 4:**
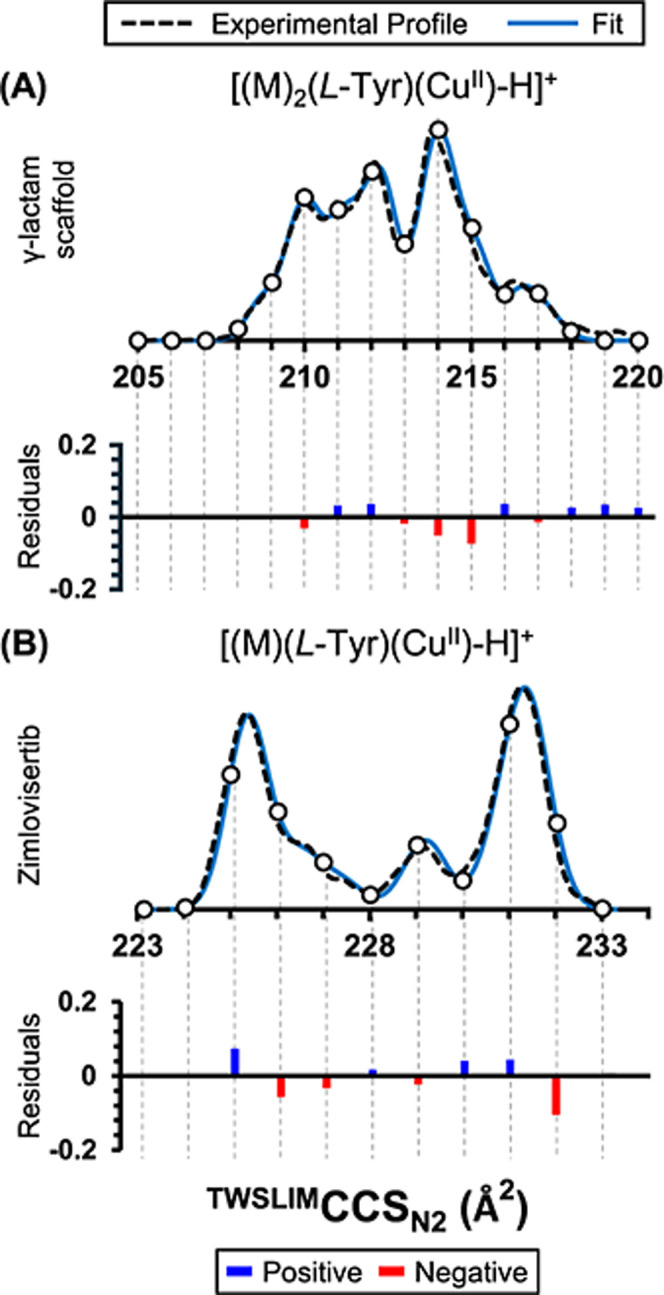
Peak fitting enables the reproduction of complex IM distributions when the CCS locations of individual components are known. Shown here are (A) the γ-lactam copper-tyrosine dimer (36 total peaks) and (B) the Zimlovisertib copper–tyrosine monomer (8 total peaks).

Predicted peak fitting of complicated ion mobility spectrometry distributions enabled us to simulate HRIM profiles for multichiral mixtures at increasingly higher R_P_ values and to ask how much R_P_ would be needed to resolve all peaks present in a mixture of all 8 stereoisomers. Collectively, we simulated R_P_ at 300, 400, 500, 750, 1000, 2000, and 3000 to generate an analytical map displaying IM peak selectively as a function of R_P_. For the copper–tyrosine monomer of Zimlovisertib, 5/8 (62%) peaks are predicted to be resolvable (10% valley) at R_P_ 300, whereas 7/8 (88%) can be resolved at R_P_ 750.^38^ However, because two peaks are extremely similar in CCS ((3*R*,4*R*,5*R*) and (3*R*,4*S*,5*R*); ΔCCS_%_ = 0.035%), resolution of all 8 peaks is achieved above R_P_ 3000 (Figure S28).

This analysis is considerably more complicated for the lactam’s copper–tyrosine dimer because there are 36 total peaks to resolve. Figure 5A depicts these simulated spectra placed into the context of a plot depicting the RP required to differentiate two peaks for any percent difference in CCS (ΔCCS_%_).^39^ At a given R_P_, a pair of peaks should be resolved if their ΔCCS_%_ is less than or equal to the corresponding value on the plot. The total number of resolved peaks is provided beside each simulated HRIM spectrum. 11/36 peaks (31%) can be resolved with a 10% valley at R_P_ 500, and 26/36 peaks (72%) are resolvable at R_P_ 3000. A relevant question is how much R_P_ would be needed to resolve all 36 features in this distribution. To address this, the total number of resolved peaks at a given R_P_ was plotted against the simulated R_P_ (Figure 5B). (Theoretical IM spectra at each simulated R_P_ are provided in Figure S29.) A power fit to this data enabled us to predict the R_P_ required to differentiate all 36 features is approximately 4730. While this level of R_P_ is currently beyond the capacity of existing commercial ion mobility spectrometry instrumentation, prototype multipass TWSLIM technology (SLIM SUPER) has demonstrated an R_P_ of nearly 2000.^40^ This would enable the separation of 7/8 peaks (88%) for the drug monomer and 24/36 peaks (66%) for the lactam dimer. We note that this analysis only considers separation in the IM dimension, and additional peak capacity can be gained with the addition of chromatography and/or energy-resolved experiments that exploited energy differences between these diastereomers. Energy-resolved studies may be particularly significant when evaluating different ion mobility separation platforms that may impart different internal energies into these complexes, resulting in differences in the observed spectra. We also note that the R_P_ recommendations provided here are specific to the copper–tyrosine complexes evaluated in this study—other complex forms incorporating different chiral selectors may impart larger differences in CCS between stereoisomers, thereby requiring a lower degree of R_P_ for baseline resolution.

**Figure 5:**
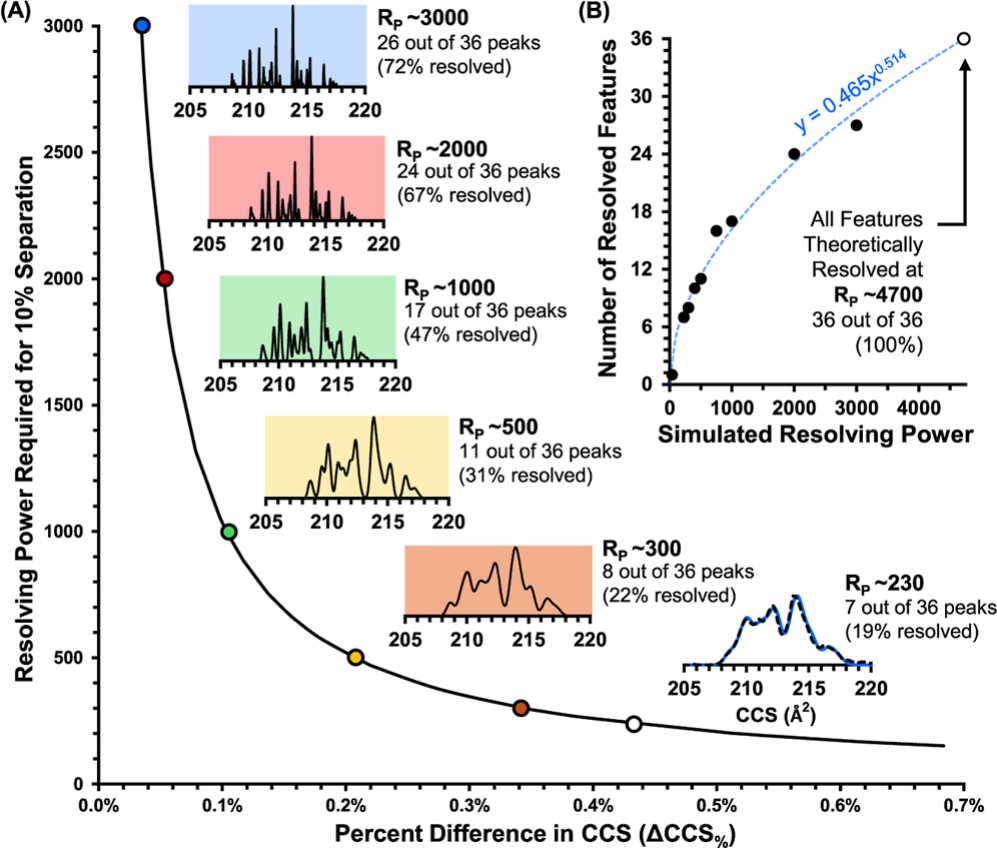
Peak fitting results are used to simulate increasing IM resolving powers. (A) γ-lactam’s [(M)_2_( *l* -Tyr)­(Cu^II^)–H]^+^ complex (36 total peaks) at an R_P_ of 230 (experimental), 300, 500, 1000, 2000, and 3000. (B) Plot relating the total number of resolved features to the simulated R_P_. A power fit predicts an R_P_ of 4730 is required to resolve all 36 features.

In conclusion, we developed an ion mobility spectrometry workflow for the analysis of multichiral center drug stereoisomers with up to 3 chiral centers by combining enantioselective copper–amino acid complexation with structurally selective HRIM instrumentation. We envision this workflow can be implemented into the pharmaceutical pipeline as a rapid assay for enantiopurity of synthetic drug products, mitigating the time and resource costs of conventional methods such as chiral LC. We also note that with a dispersion time of <1 s, TWSLIM operates on a time scale amenable to incorporation into LC–MS workflows, opening the door for chiral analysis using multidimensional LC-TWSLIM-MS. Complexation using copper–tyrosine imparted CCS differences between 0.08% and 1.43%, enabling the resolution of 7 out of 8 (88%) enantiomer pairs evaluated in this study. Using a copper–tyrosine-bound monomer of the full drug, we achieved differentiation of 4/8 features in a mixture of all 8 stereoisomers (R_P_ 214), and the majority of these features (7/8) can be resolved at an R_P_ of 750. The dimer-incorporated copper–tyrosine complex yielded unexpectedly high differences in CCS on individually analyzed stereoisomers of the γ-lactam scaffold (as high as 1.43%). However, because the number of heterodimers increases as a function of the number of isomers in a mixture, the dimer-incorporated complex also imparted spectral complexity, which made differentiation of a mixture of 8 stereoisomers particularly challenging. These results underscore the complexity of chiral separations and the versatility of HRIM instrumentation as a tool for enantiomer analysis.

## Supplementary Material



## Notes

Details on chemicals and reagents used in this study (structures, stereochemistry, and vendor sources), NMR characterization for Pfizer reagents, instrument schematics, summary of ^DT^CCS_N2_ and ^TWSLIM^CCS_N2_ measurements, and summary of peak fitting statistics (PDF)

## Abbreviations

2D-LC: two-dimensional liquid chromatography
API: active pharmaceutical ingredient
^DT^CCS_N2_: collision cross-section measured in nitrogen buffer gas on a drift tube platform
CD: circular dichroism spectroscopy
cIMS: cyclic ion mobility spectrometry
HRIM: high-resolution ion mobility spectrometry
IM: ion mobility spectrometry
IM-MS: ion mobility–mass spectrometry
LOD: limit of detection
*m*/*z*: mass-to-charge ratio
MS: mass spectrometry
NMR: nuclear magnetic resonance spectroscopy
R_P_: resolving power
RPLC: reversed-phase liquid chromatography
R_P‑P_: two-peak resolution
TIMS: trapped ion mobility spectrometry
TWSLIM: traveling wave structures for lossless ion manipulations
Tyr: tyrosine
ΔCCS_%_: percent difference in CCS.
