# Supplementary material for: Rapid Chiral Analysis of Drugs with Multiple Stereocenters via High-Resolution Ion Mobility
Source: Anal Chem. 2026 Jul 29;98(31):22627–35. doi: 10.1021/acs.analchem.6c03422 (PMC13470978; doi:10.1021/acs.analchem.6c03422)
Supplement: Supplementary file 1 [file ac6c03422_si_001.pdf]

Supporting Information

Rapid chiral analysis of drugs with multiple stereocenters via high-resolution ion mobility

Benjamin K. Blakley[a], Jody C. May[a], Valeria Guidolin[b], Bao Nguyen[b] and John A. McLean[a]*

[a] Department of Chemistry, Center for Innovative Technology, Vanderbilt Institute of Chemical Biology, Vanderbilt-Ingram Cancer Center, and Vanderbilt Institute for Integrated Biosystems Research, Vanderbilt University, Nashville, TN 37235-1822 (USA)

[b] Pfizer, Inc., PharmSci Small Molecules, Analytical Research and Development, Groton, CT (USA)

*Corresponding author email: john.a.mclean@vanderbilt.edu

Table of Contents

SI Content	Description	Page
**Text S1**	Peak analysis details	S3
**Table S1**	Reagents and vendor information	S4
**Figure S1**	Analyte structures and stereochemistry	S5
**Figure S2**	NMR spectrum of PF-06772169	S6
**Figure S3**	NMR spectrum of PF-06691428	S7
**Figure S4**	NMR spectrum of PF-06839064	S8
**Figure S5**	NMR spectrum of PF-07037226	S9
**Figure S6**	NMR spectrum of PF-07037224	S10
**Figure S7**	NMR spectrum of PF-07037222	S11
**Figure S8**	NMR spectrum of PF-07037232	S12
**Figure S9**	NMR spectrum of PF-07037238	S13
**Figure S10**	NMR spectrum of PF-06687838	S14
**Figure S11**	NMR spectrum of PF-06800360	S15
**Figure S12**	NMR spectrum of PF-06868653	S16
**Figure S13**	NMR spectrum of PF-06868650	S17
**Figure S14**	NMR spectrum of PF-06730079	S18
**Figure S15**	NMR spectrum of PF-06868651	S19
**Figure S16**	NMR spectrum of PF-06868654	S20
**Figure S17**	NMR spectrum of PF-06650833	S21
**Figure S18**	Instrument schematics	S22
**Figure S19**	DTIMS-MS heatmap: γ-lactam scaffold + copper (II) acetate + *L*-tyrosine	S23
**Figure S20**	DTIMS-MS heatmap: full drug + copper (II) acetate + *L*-tyrosine	S24
**Table S2**	$^{DT}CCS_{N2}$ measurements for target complexes	S25
**Table S3**	Interday CCS reproducibility for the two IM instruments	S26
**Figure S21**	DTIMS separation achieved for ternary copper complexes of interest	S27
**Figure S22**	TWSLIM-MS heatmap: γ-lactam scaffold + copper (II) acetate + *L*-tyrosine	S28
**Figure S23**	TWSLIM-MS heatmap: full drug + copper (II) acetate + *L*-tyrosine	S29
**Figure S24**	Preliminary quantitation experiments for binary mixture of enantiomers	S30
**Figure S25**	CID fragmentation pathways for the γ-lactam $[(M)_2(L\text{-Tyr})(Cu^{II})\text{-H}]^+$	S31
**Figure S26**	TWSLIM spectra for the γ-lactam scaffold's $[(M)_2(L\text{-Tyr})(Cu^{II})\text{-H}]^+$ complex: binary mixtures of diastereomers	S32
**Figure S27**	Illustration of residual analysis to assess peak fitting	S33
**Table S4**	Peak statistics obtained via peak fitting	S34
**Figure S28**	Simulated heightened resolving powers: full drug's $[(M)(L\text{-Tyr})(Cu^{II})\text{-H}]^+$	S35
**Figure S29**	Simulated heightened resolving powers: γ-lactam scaffold's $[(M)_2(L\text{-Tyr})(Cu^{II})\text{-H}]^+$	S36

S1. Peak Analysis Details

Peak Metrics

Resolving power (R_P) describes the width of a single peak and is defined as the CCS divided by the width of the peak at half the maximum height.

$$R_P = \frac{CCS}{FWHM} \qquad (1)$$

To ensure highly accurate peak metrics, R_P was calculated using a peak fitting workflow in Microsoft Excel.

Peak Fitting

Peak fitting for TWSLIM data was performed in Microsoft Excel. For each feature in extracted experimental distributions, a Gaussian was generated whose μ (mean) was assigned based on the centroid of experimental features. σ (standard deviations) were assigned based on visual alignments of the generated Gaussians with experimental data. This allowed for calculation of resolving power (R_P) for each peak, as shown in Equation S2:

$$R_P = \frac{\mu}{2\sqrt{2ln2} * \sigma} (2)$$

To reconstruct IM distributions, peak fitting was performed to obtain μ and σ values for each feature in a given distribution (for both homodimers and heterodimers, where applicable). For heterodimers which did not directly resolve, the μ was approximated by taking the average of the CCS values from the homodimers of the two individual stereoisomers comprising the heterodimer, while the σ was assigned based on the average resolving power for resolved peaks. Initially, each Gaussian was assigned the intensity of its corresponding experimental feature and normalized to the highest intensity among all experimental values.

To generate a theoretical composite IM distribution, the individual Gaussians were point aligned, then summed across the entire CCS distribution. To assess the validity of the predicted IM distribution, residuals were calculated at each point using Equation S3:

$$Residual = y - \hat{y} \qquad (3)$$

where y is the normalized experimental intensity at a given CCS and $\hat{y}$ is the predicted normalized intensity. To optimize peak fits for theoretical IM distributions, intensities of each individual Gaussian were then adjusted such that the residual was minimized at each CCS in the distribution.

To simulate experimental measurements at increasingly high resolving powers, σ values for each individual Gaussians that compose the mixture distribution were simultaneously scaled until a desired average resolving power was reached.

Table S1. List of reagents used in this study.

				Molecular Formula	Exact Mass (Da)	Pfizer #	Corresponding Publication
γ-lactam scaffold	(3*S*, 4*S*, 5*S*)		*SSS*	$C_7H_{12}FNO_2$	161.09	PF-06691428	Lee, K.L.; Ambler, C.M.; Anderson, D.R. *et al. J. Med. Chem.* **2017**, 60, 13, 5521-5542. **DOI:** 10.1021/acs.jmedchem.7b00231
	(3*R*, 4*R*, 5*R*)		*RRR*			PF-06839064	
	(3*R*, 4*S*, 5*S*)		*RSS*			PF-06772169	
	(3*S*, 4*R*, 5*R*)		*SRR*			PF-07037226	
	(3*S*, 4*R*, 5*S*)		*SRS*			PF-07037224	
	(3*R*, 4*S*, 5*R*)		*RSR*			PF-07037238	
	(3*R*, 4*R*, 5*S*)		*RRS*			PF-07037222	
	(3*S*, 4*S*, 5*R*)		*SSR*			PF-07037232	
Full drug	(3*S*, 4*S*, 5*S*)		*SSS*	$C_{18}H_{20}FN_3O_4$	361.14	PF-06650833	
	(3*R*, 4*R*, 5*R*)		*RRR*			PF-06800360	
	(3*R*, 4*S*, 5*S*)		*RSS*			PF-06687838	
	(3*S*, 4*R*, 5*R*)		*SRR*			PF-06868653	
	(3*S*, 4*R*, 5*S*)		*SRS*			PF-06868650	
	(3*R*, 4*S*, 5*R*)		*RSR*			PF-06868654	
	(3*R*, 4*R*, 5*S*)		*RRS*			PF-06730079	
	(3*S*, 4*S*, 5*R*)		*SSR*			PF-06868651	

		Molecular Formula	Exact Mass (Da)	PubChem ID	Vendor Source (Product #)
Copper (II) acetate	Cu^{II}	$C_4H_6CuO_4$	180.96	8895	Sigma (326755)
L-Tyrosine	*L*-Tyr	$C_9H_{11}NO_3$	181.07	6057	Sigma (T8909)
D-Tyrosine	*D*-Tyr			71098	Alfa Aesar (A17709)

Figure S1. Structures for all 16 stereoisomers (8 per analyte) used in this work.

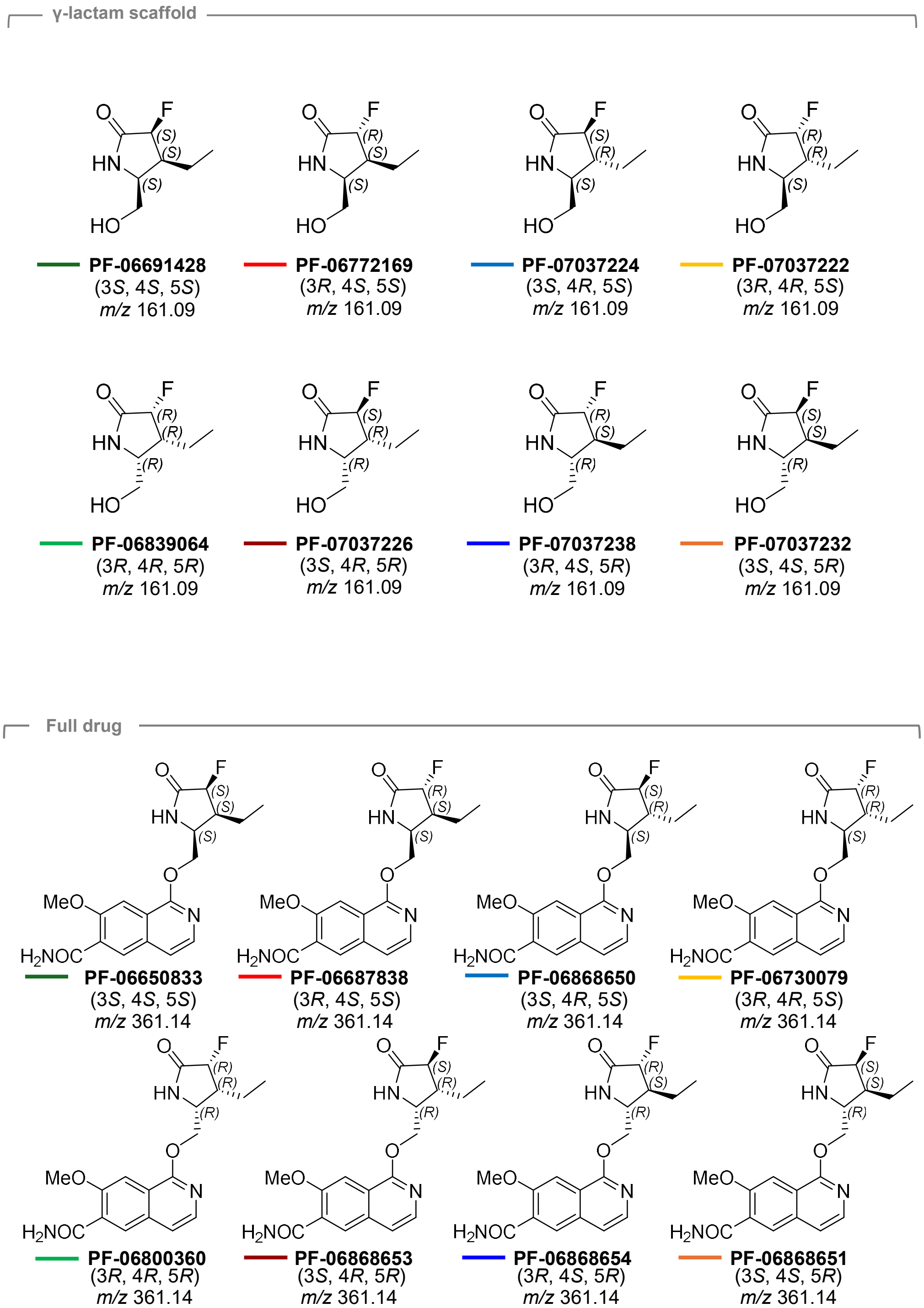

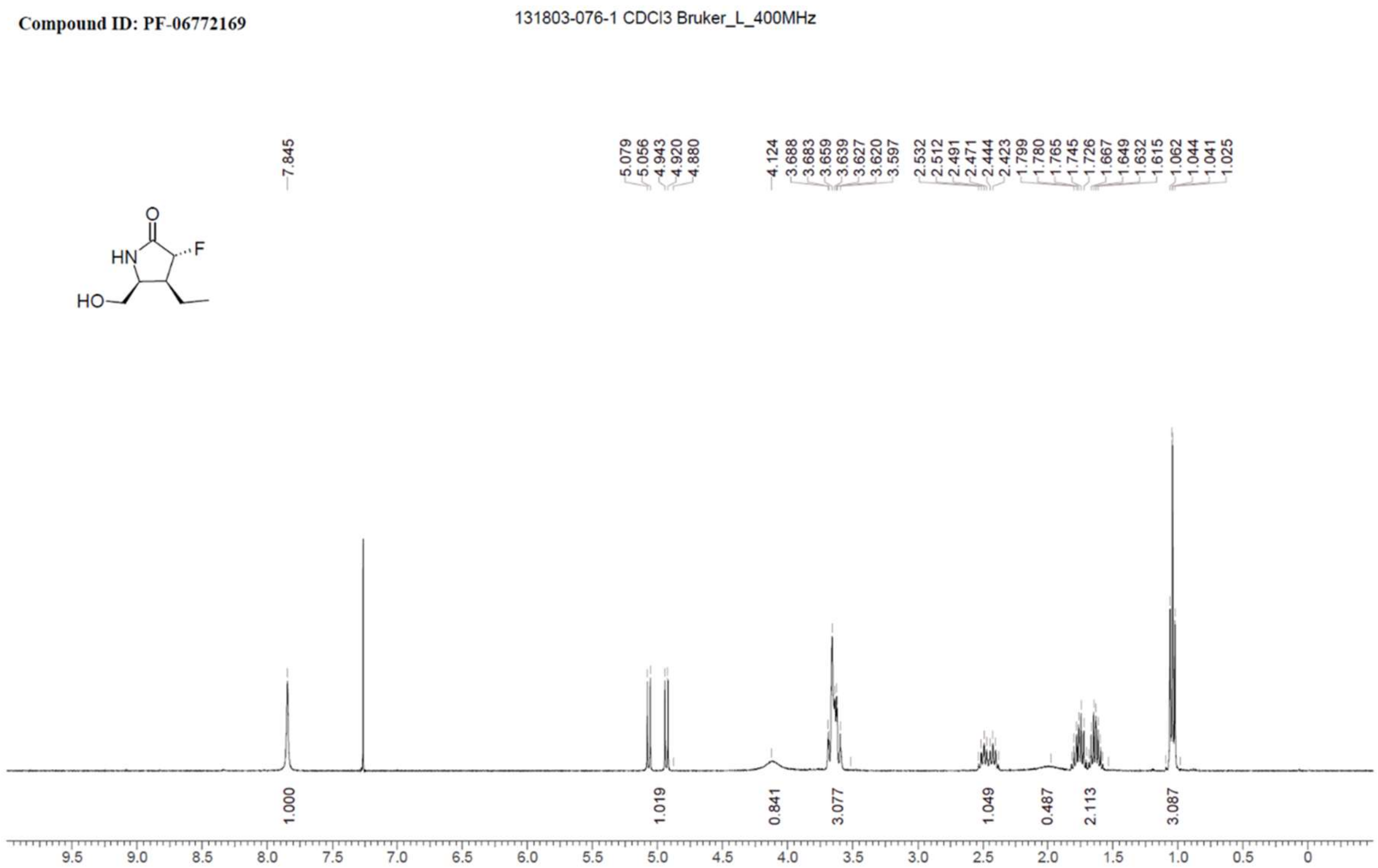

Figure S2. NMR Spectra PF-06772169 [γ-lactam, (3*R*, 4*S*, 5*S*)].

Compound ID: PF-06691428 in DMSO-d_6, 600MHz

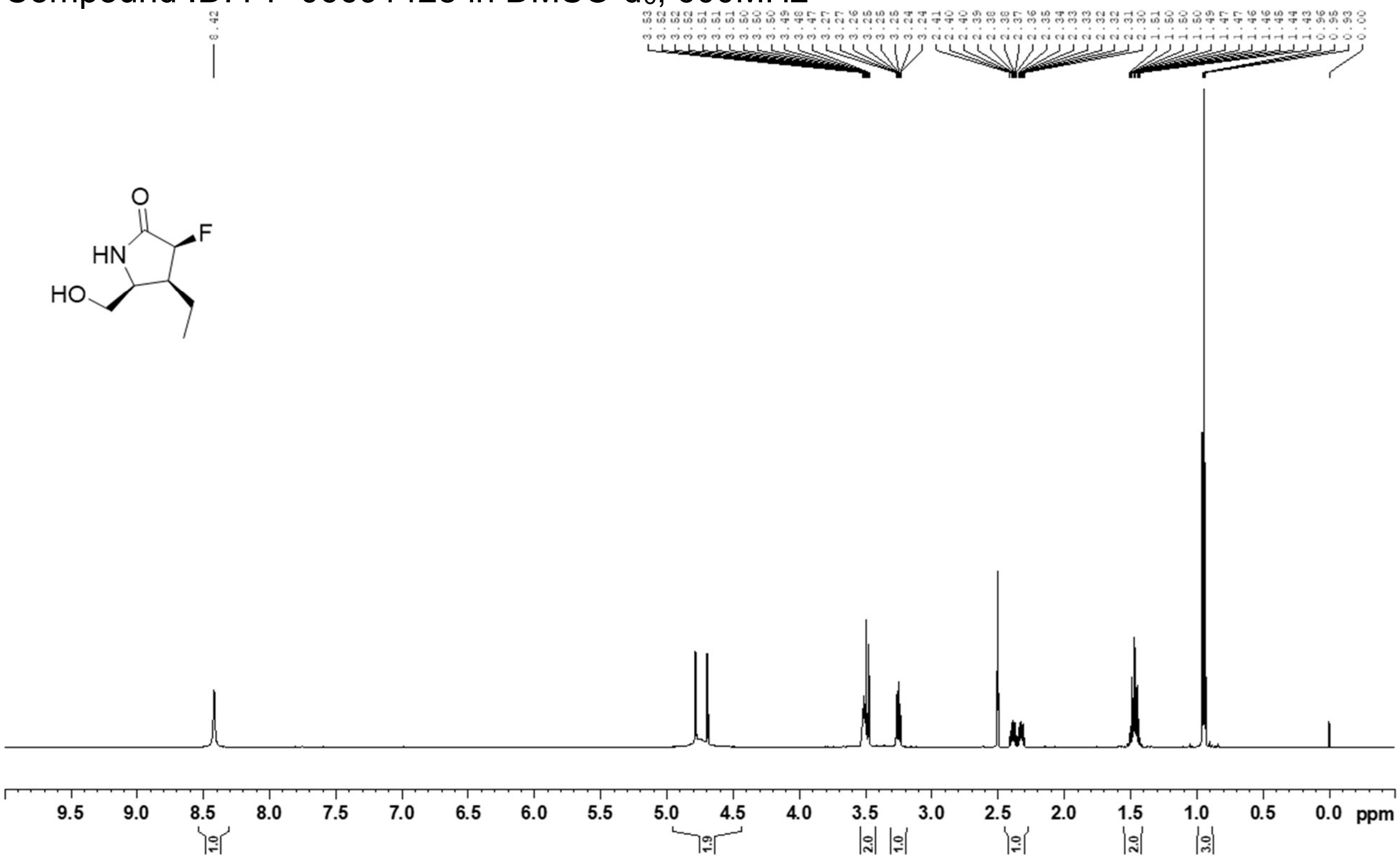

Figure S3. NMR Spectra PF-06691428 [γ-lactam, (3*S*, 4*S*, 5*S*)].

Compound ID: PF-06839064 in DMSO-d_6, 600MHz

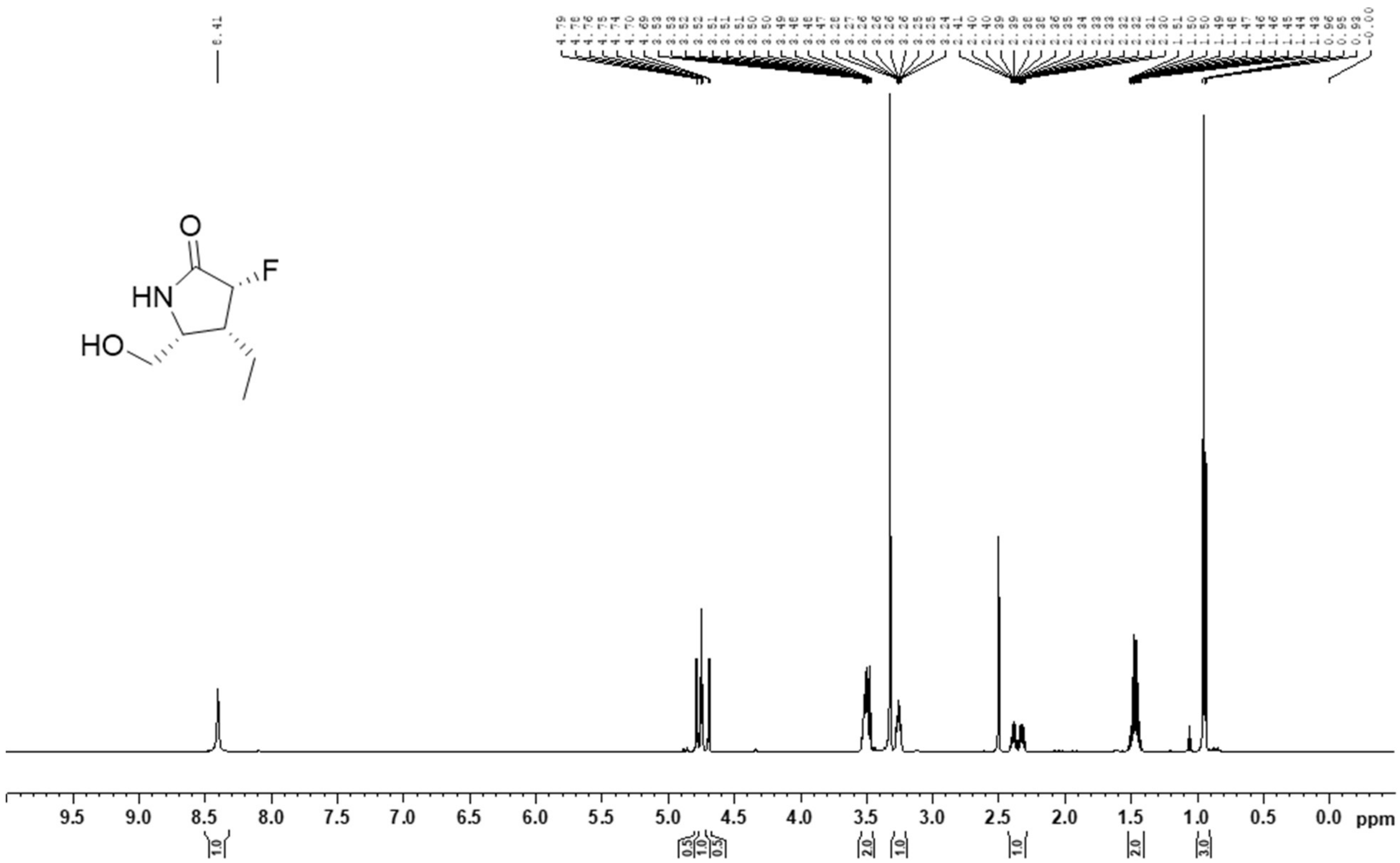

Figure S4. NMR Spectra PF-06839064 [γ-lactam, (3*R*, 4*R*, 5*R*)].

Compound ID: PF-07037226 in DMSO-d_6, 600MHz

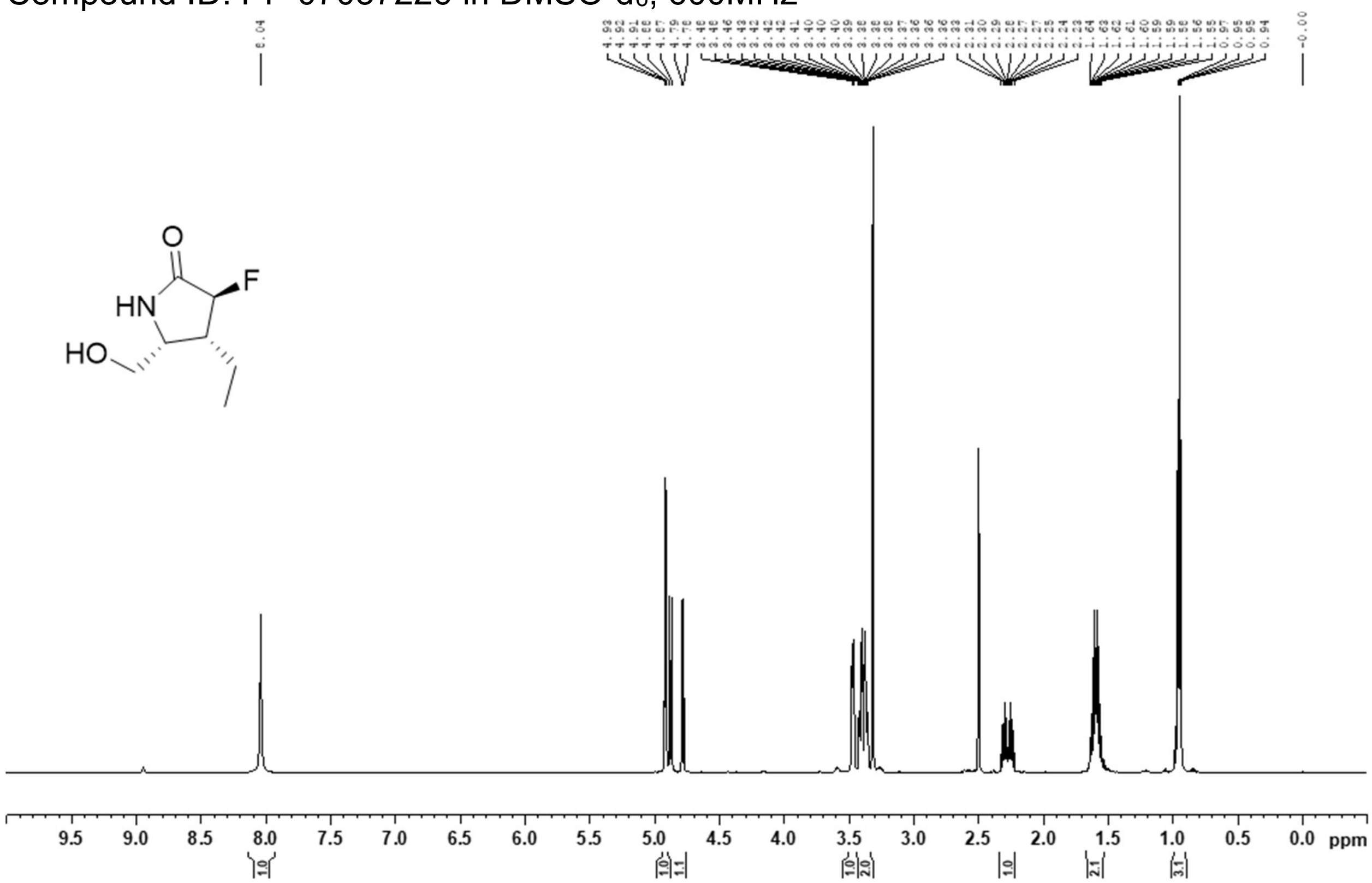

Figure S5. NMR Spectra PF-07037226 [γ-lactam, (3*S*, 4*R*, 5*R*)].

Compound ID: PF-07037224 in DMSO-d_6, 600MHz

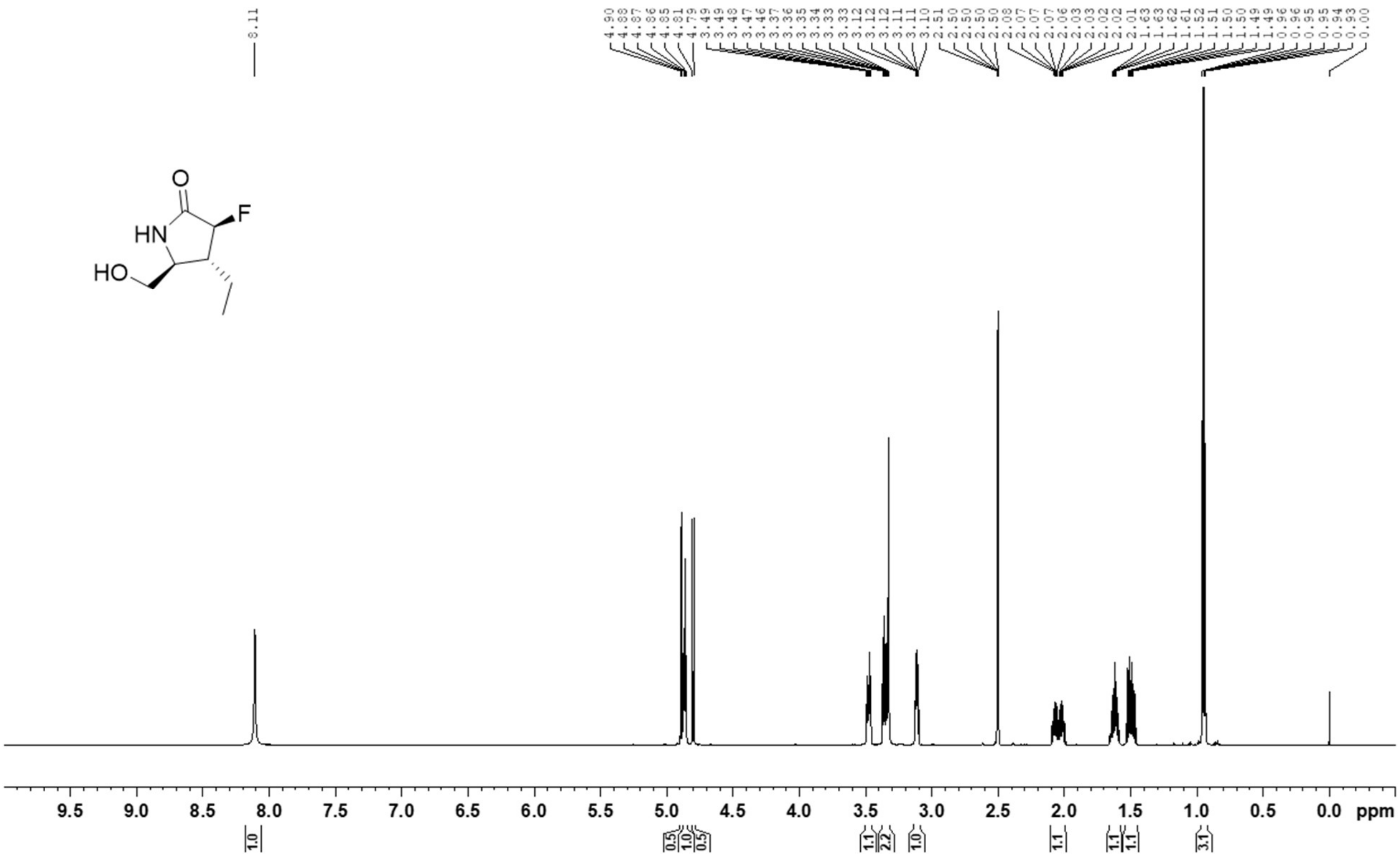

Figure S6. NMR Spectra PF-07037224 [γ-lactam, (3*S*, 4*R*, 5*S*)].

Compound ID: PF-07037222 in DMSO-d_6, 600MHz

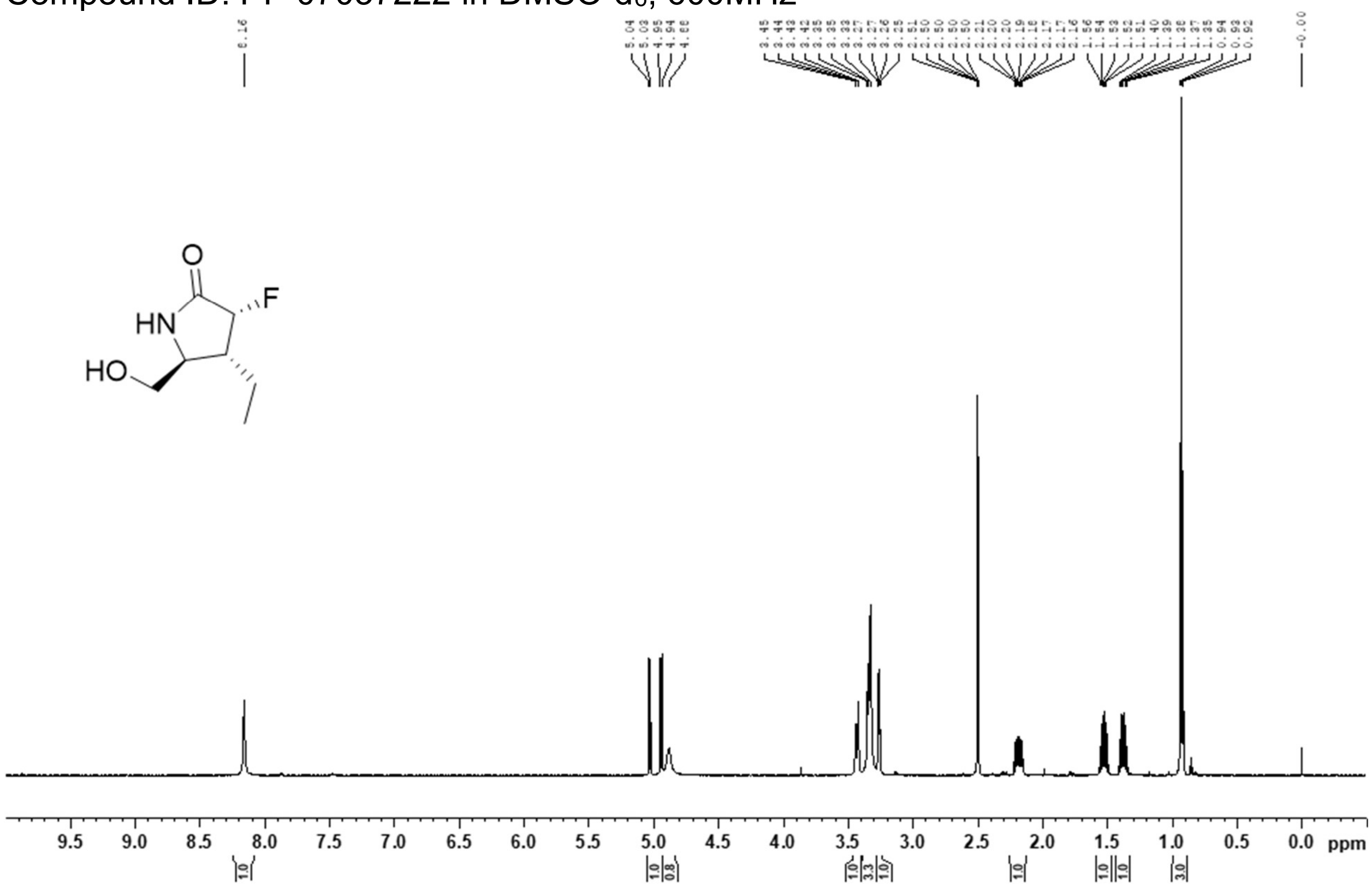

Figure S7. NMR Spectra PF-07037222 [γ-lactam, (3*R*, 4*R*, 5*S*)].

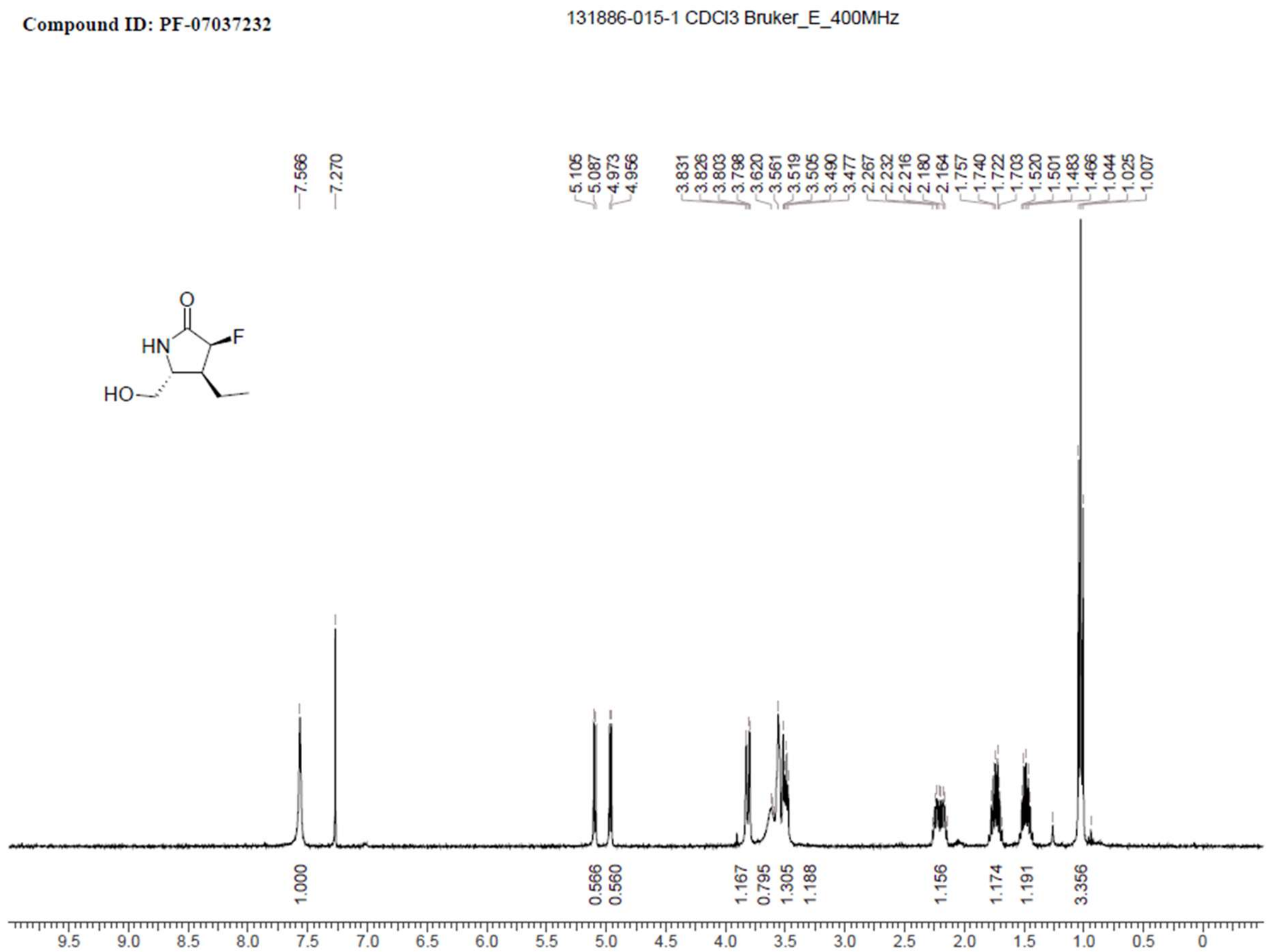

Figure S8. NMR Spectra PF-07037232 [γ-lactam, (3*S*, 4*S*, 5*R*)].

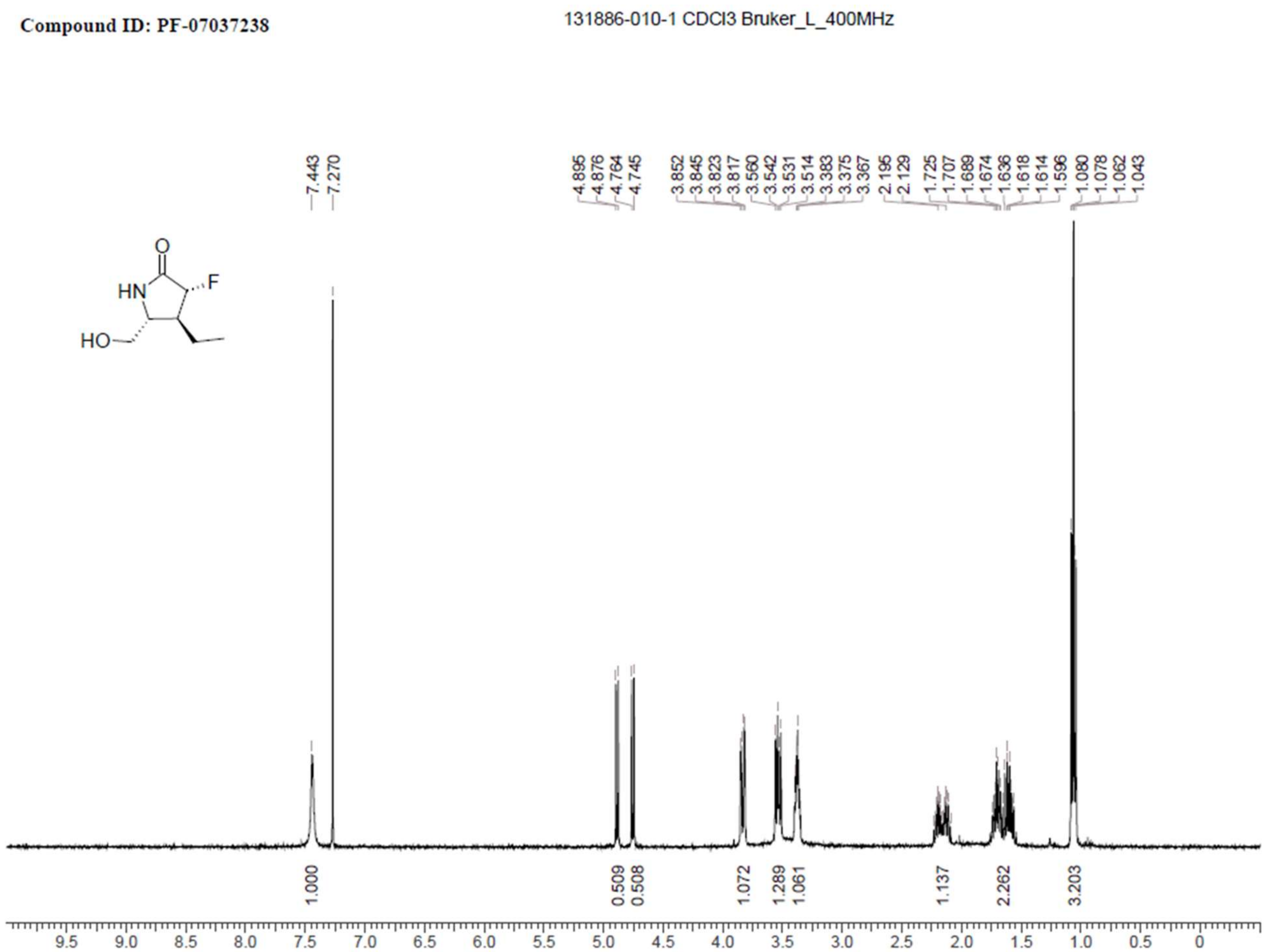

Figure S9. NMR Spectra PF-07037238 [γ-lactam, (3*R*, 4*S*, 5*R*)].

Compound ID: PF-06687838 in DMSO-d$_6$, 600MHz

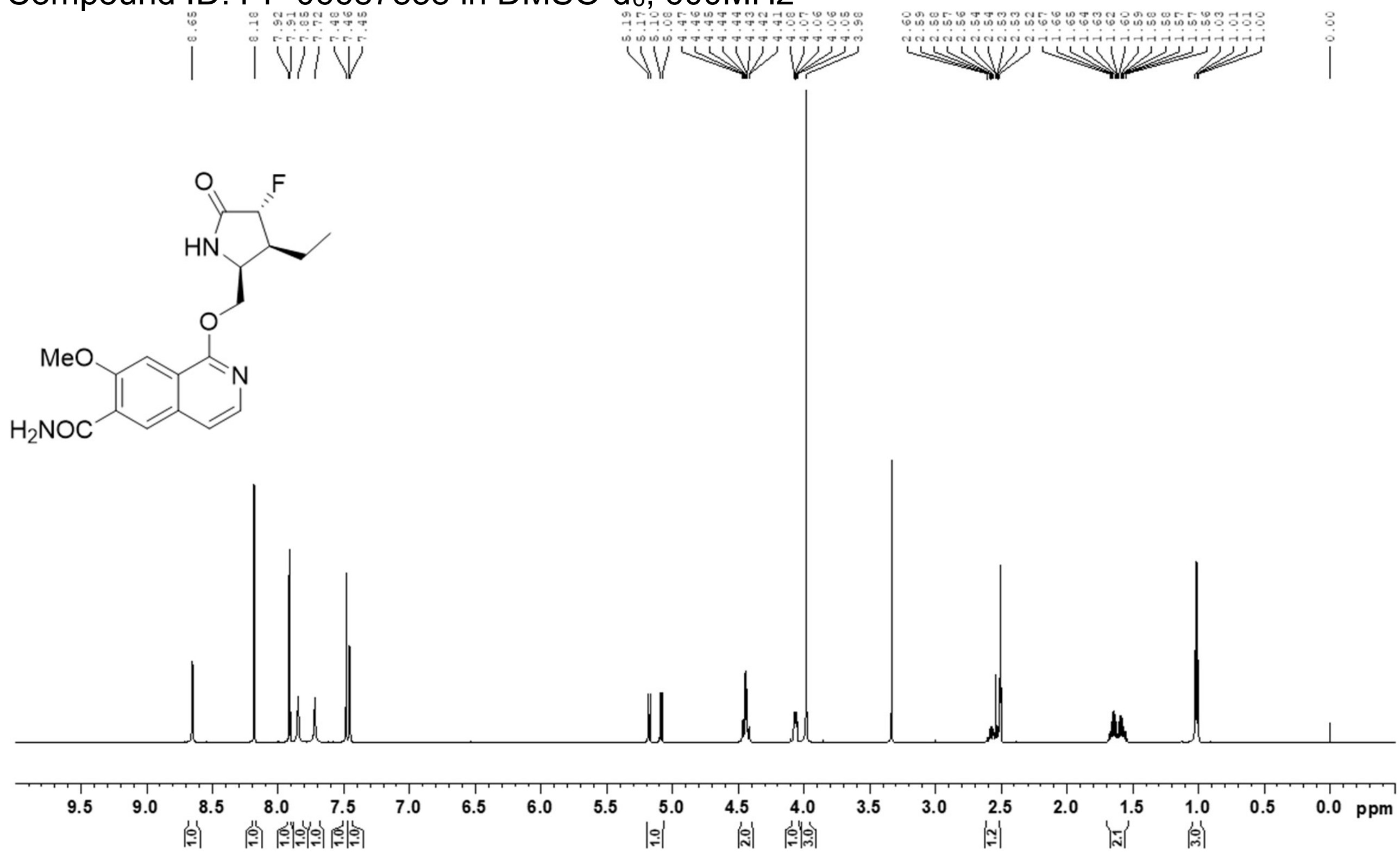

Figure S10. NMR Spectra PF-06687838 [Zimlovisertib (full drug), (3*R*, 4*S*, 5*S*)].

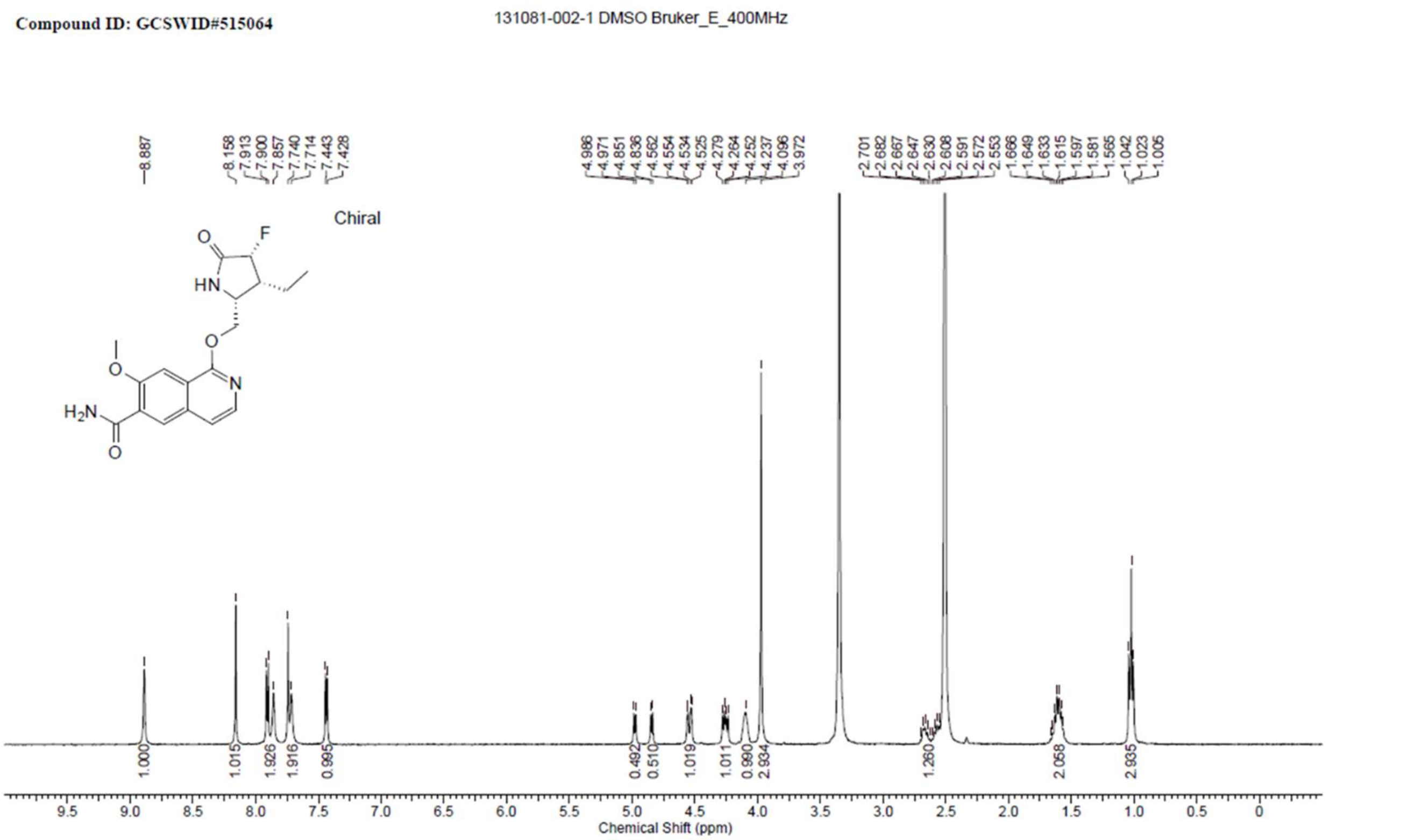

Figure S11. NMR Spectra PF-06800360 [Zimlovisertib (full drug), (3*R*, 4*R*, 5*R*)].

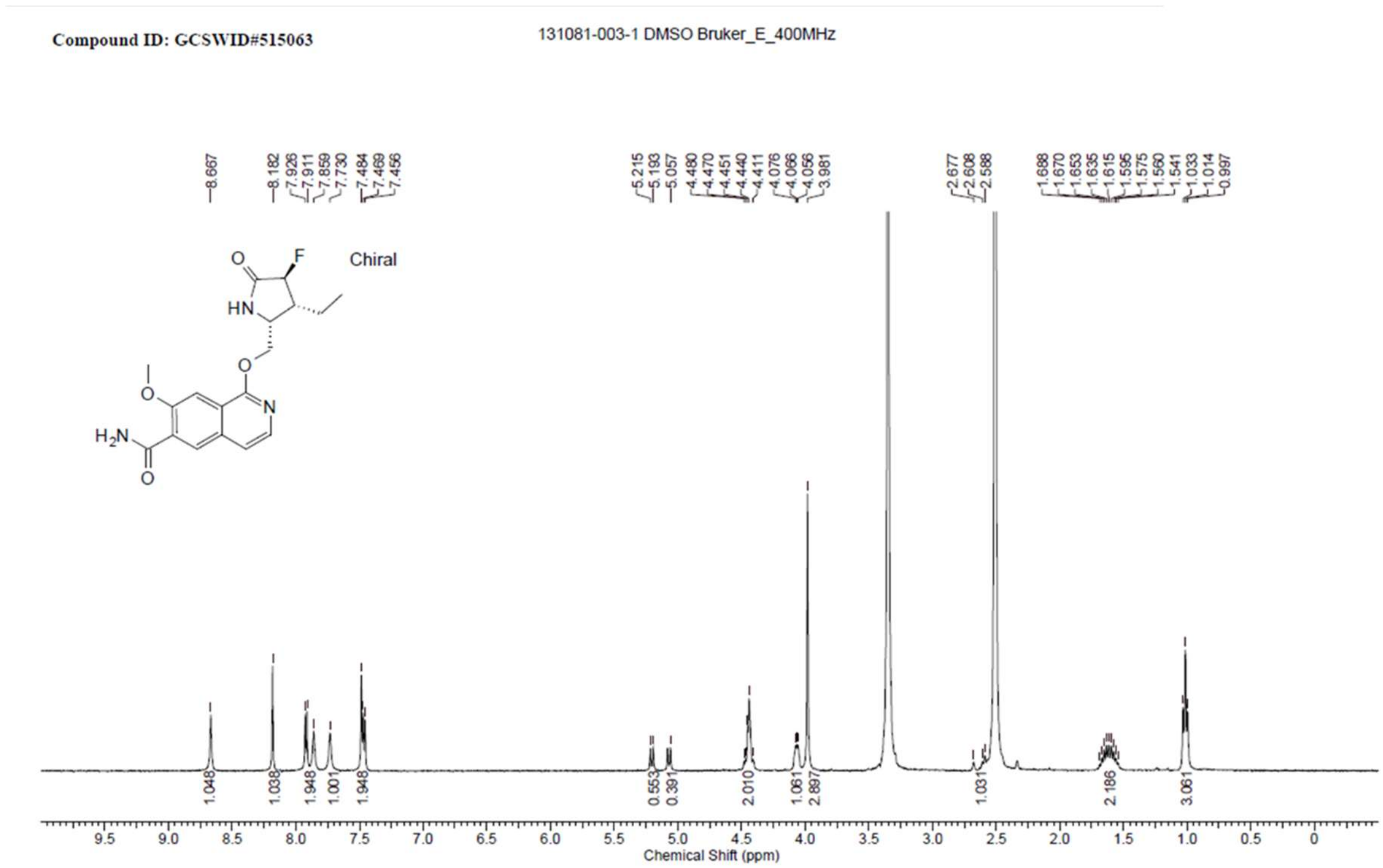

Figure S12. NMR Spectra PF-06868653 [Zimlovisertib (full drug), (3*S*, 4*R*, 5*R*)].

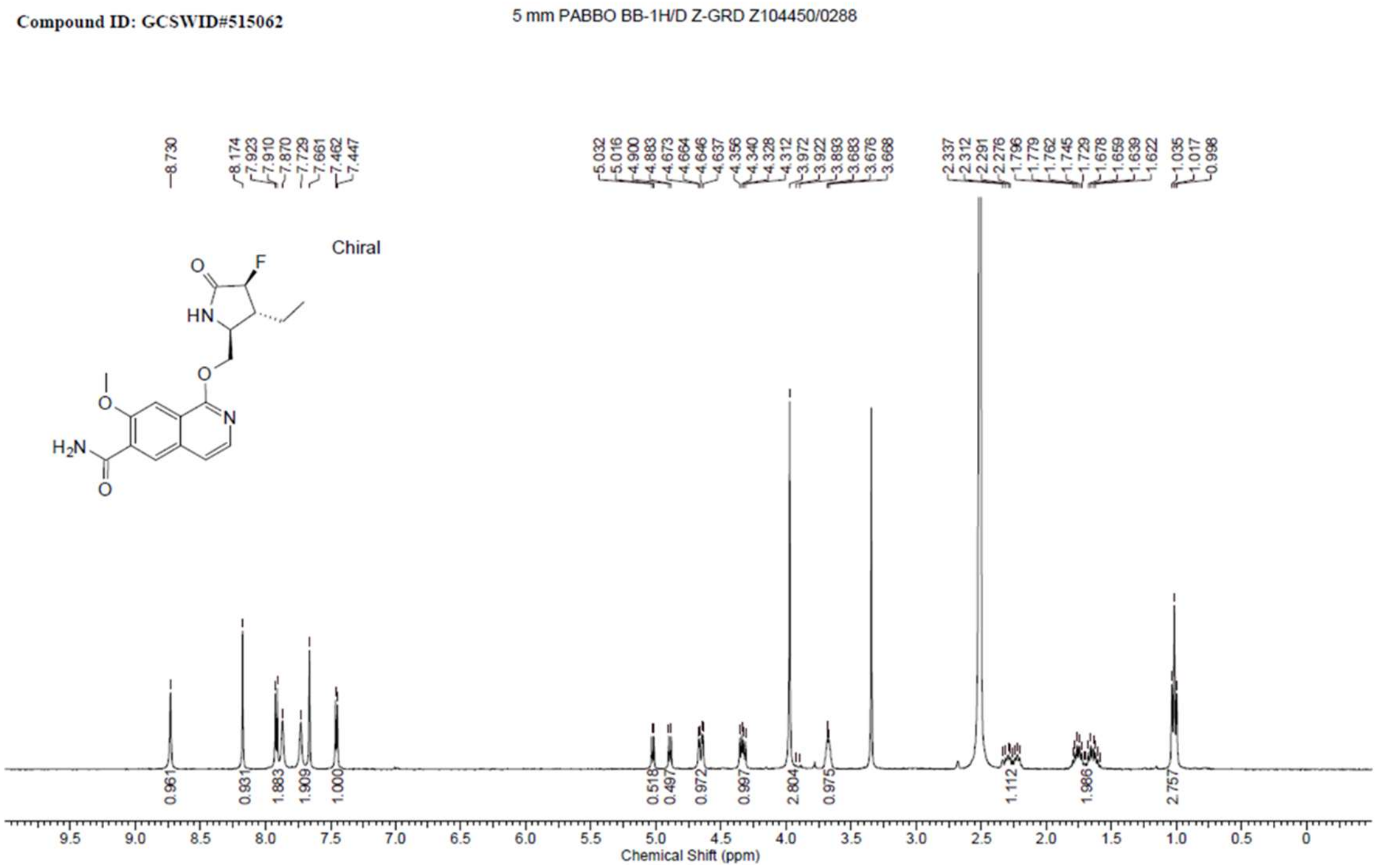

Figure S13. NMR Spectra PF-06868650 [Zimlovisertib (full drug), (3*S*, 4*R*, 5*S*)].

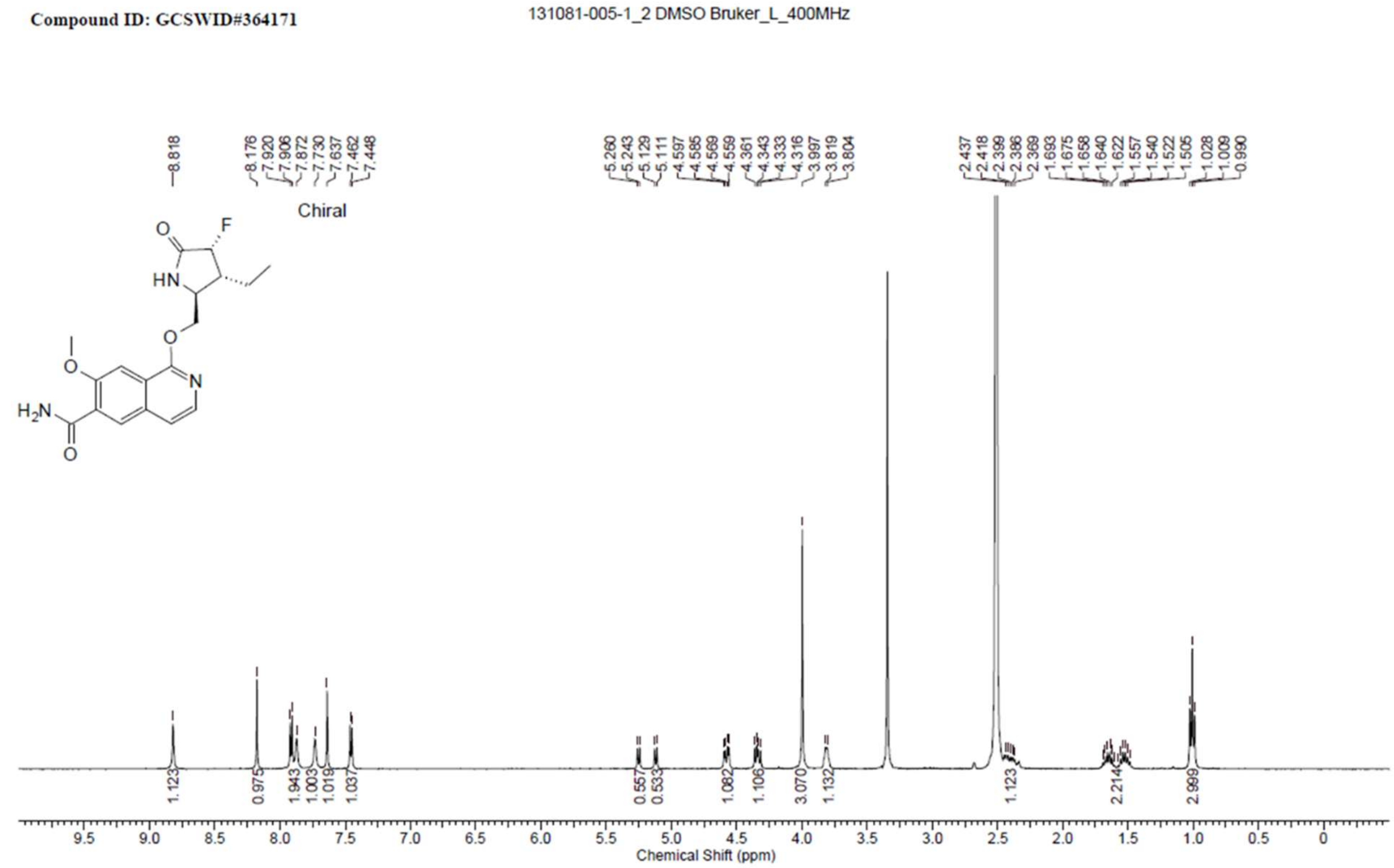

Figure S14. NMR Spectra PF-06730079 [Zimlovisertib (full drug), (3*R*, 4*R*, 5*S*)].

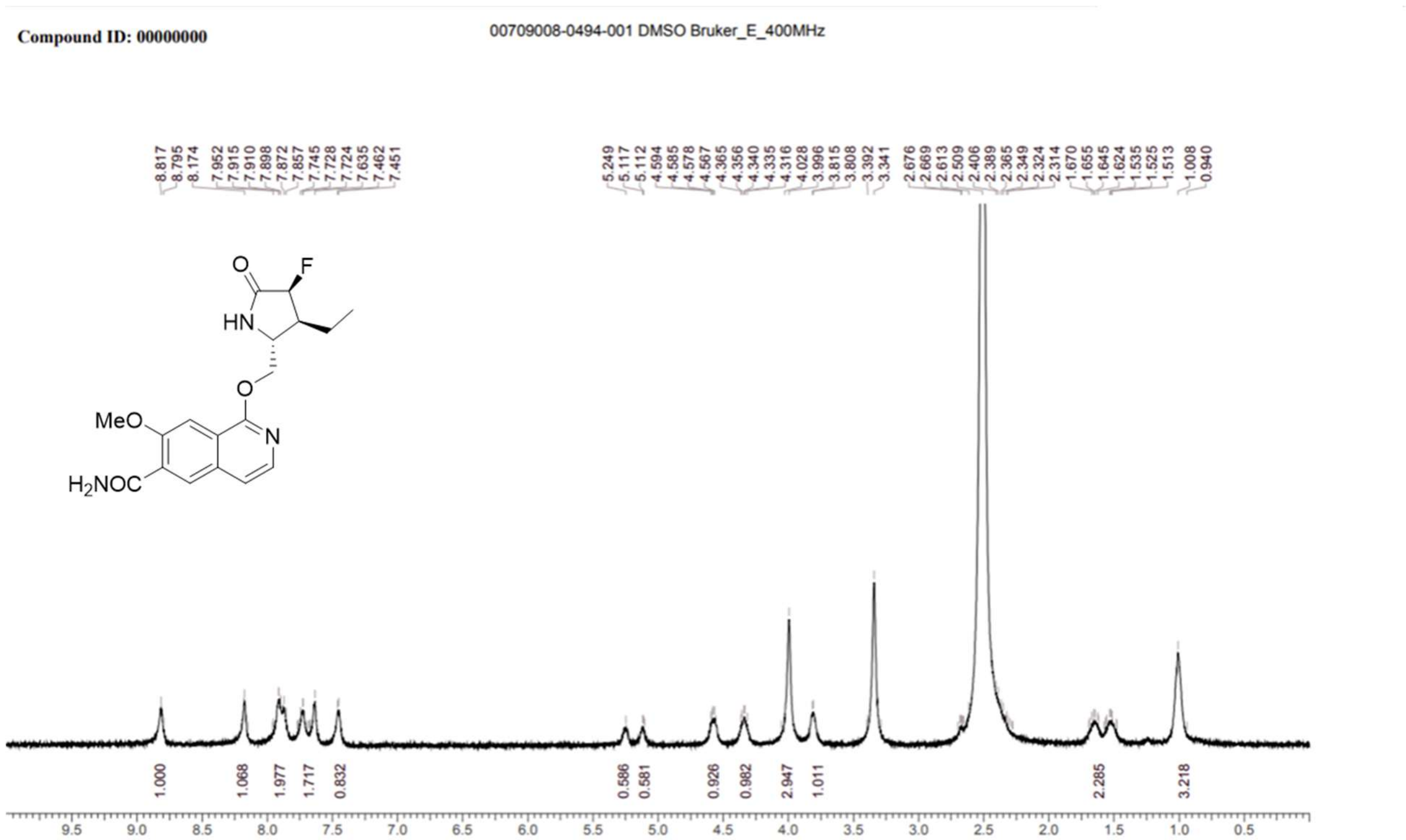

Figure S15. NMR Spectra PF-06868651 [Zimlovisertib (full drug), (3*S*, 4*S*, 5*R*)].

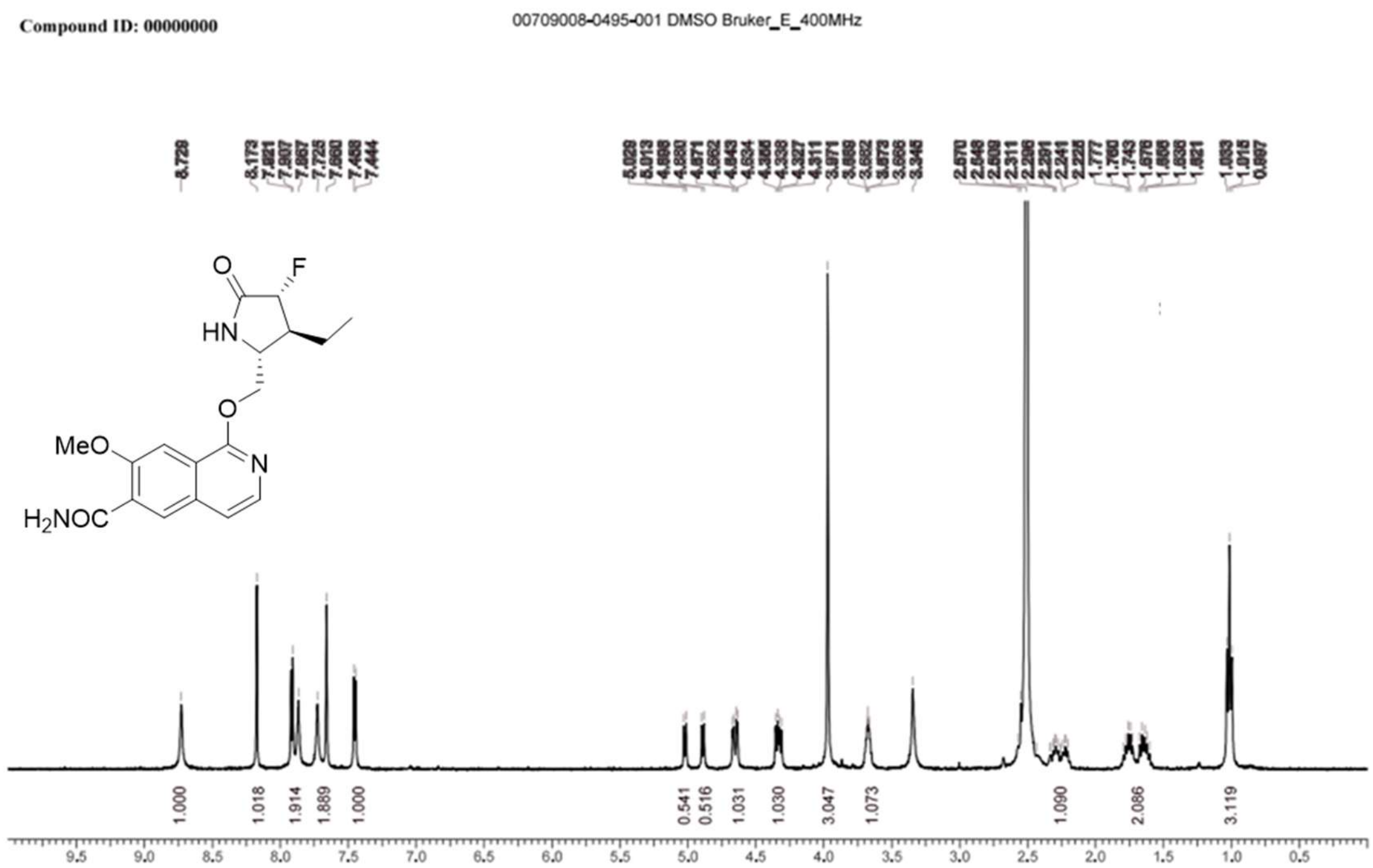

Figure S16. NMR Spectra PF-06868654 [Zimlovisertib (full drug), (3*R*, 4*S*, 5*R*)].

Compound ID: PF-06650833 in DMSO-d$_6$, 600MHz

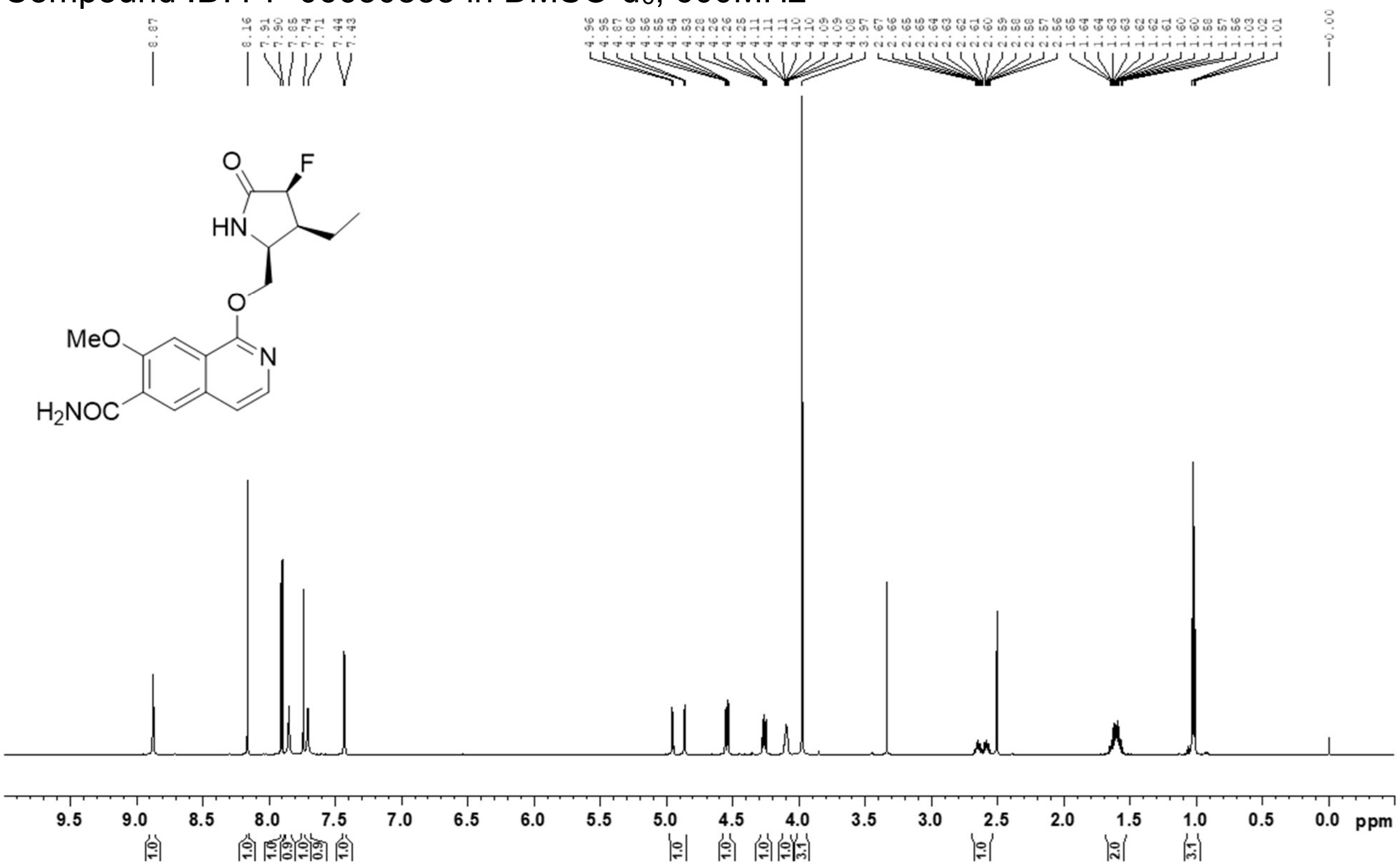

Figure S17. NMR Spectra PF-06650833 [Zimlovisertib (full drug), (3*S*, 4*S*, 5*S*)].

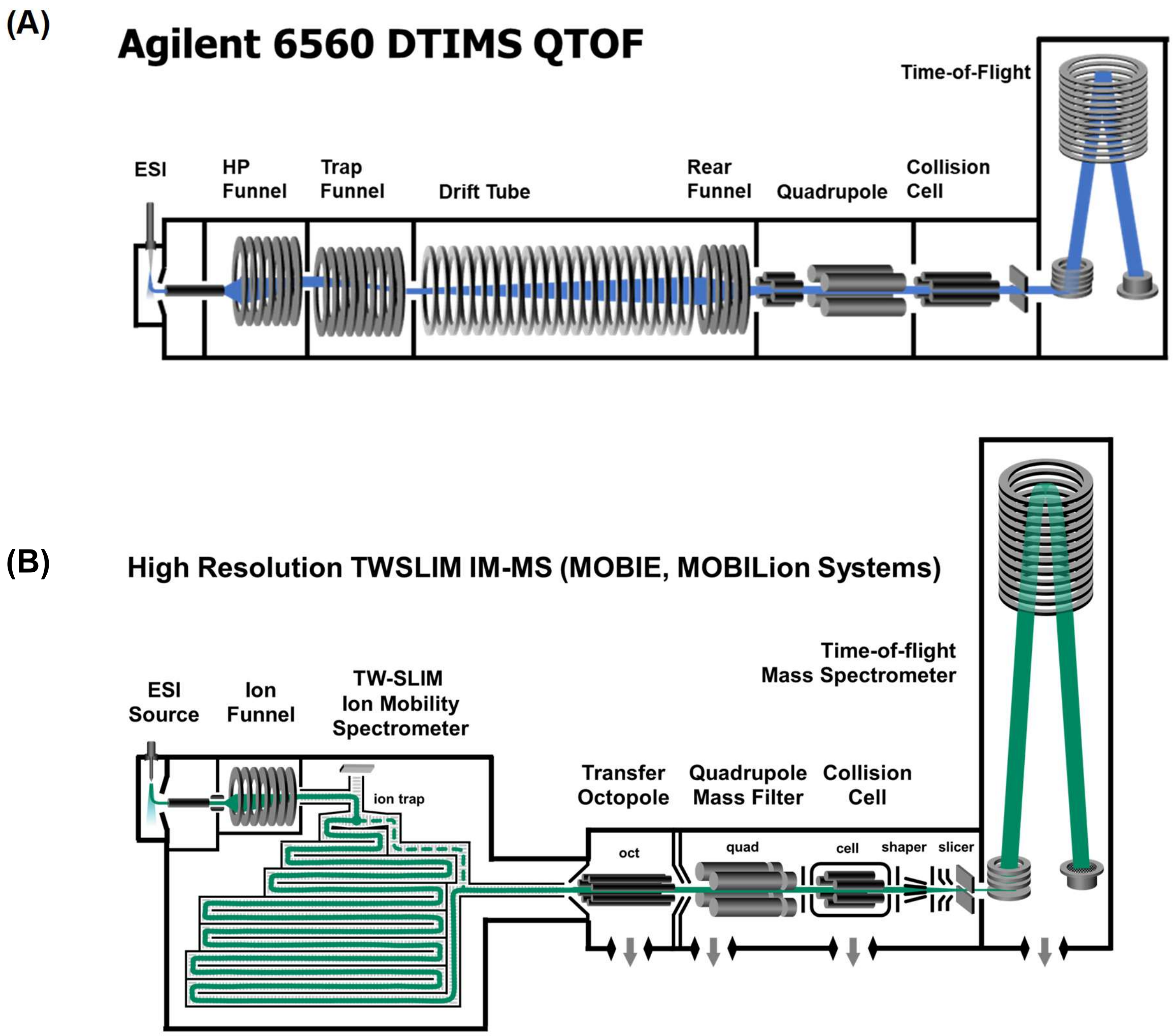

Figure S18. Instrument schematics for ion mobility-mass spectrometers used in the study. **(A)** An Agilent 6560 DTIMS-QTOF instrument with an IM resolving power of approximately 50. **(B)** A MOBILion MOBIE SLIM-based HR-TWIMS instrument with an IM resolving power of approximately 200. The MOBIE is coupled to an Agilent 6546 QTOF.

DTIMS: 20 µM γ-lactam scaffold (3*S*,4*R*,5*S*) + *L*-Tyrosine + $Cu(OAc)_2$

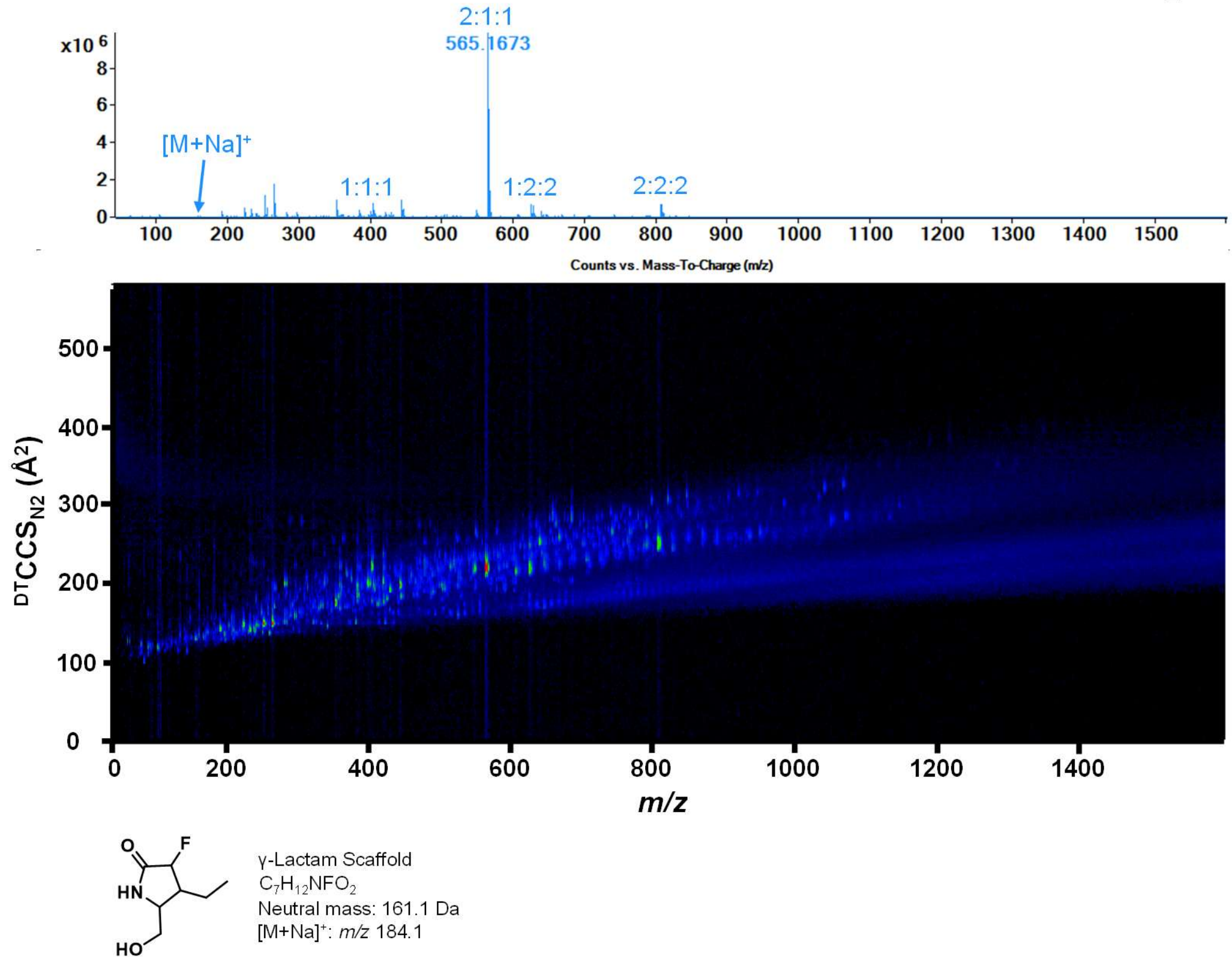

Figure S19. DTIMS-MS spectrum for the γ-lactam scaffold with copper (II) acetate and *L*-tyrosine (all 20 µM in 1:1 methanol:water). Here, ratios listed above peaks indicate the stoichiometry of the corresponding drug:tyrosine:copper complex. For instance, a 1:2:2 annotation indicates a complex consisting of 1 analyte ligand, 2 tyrosine chiral reference ligands, and 2 copper ions (a binuclear complex).

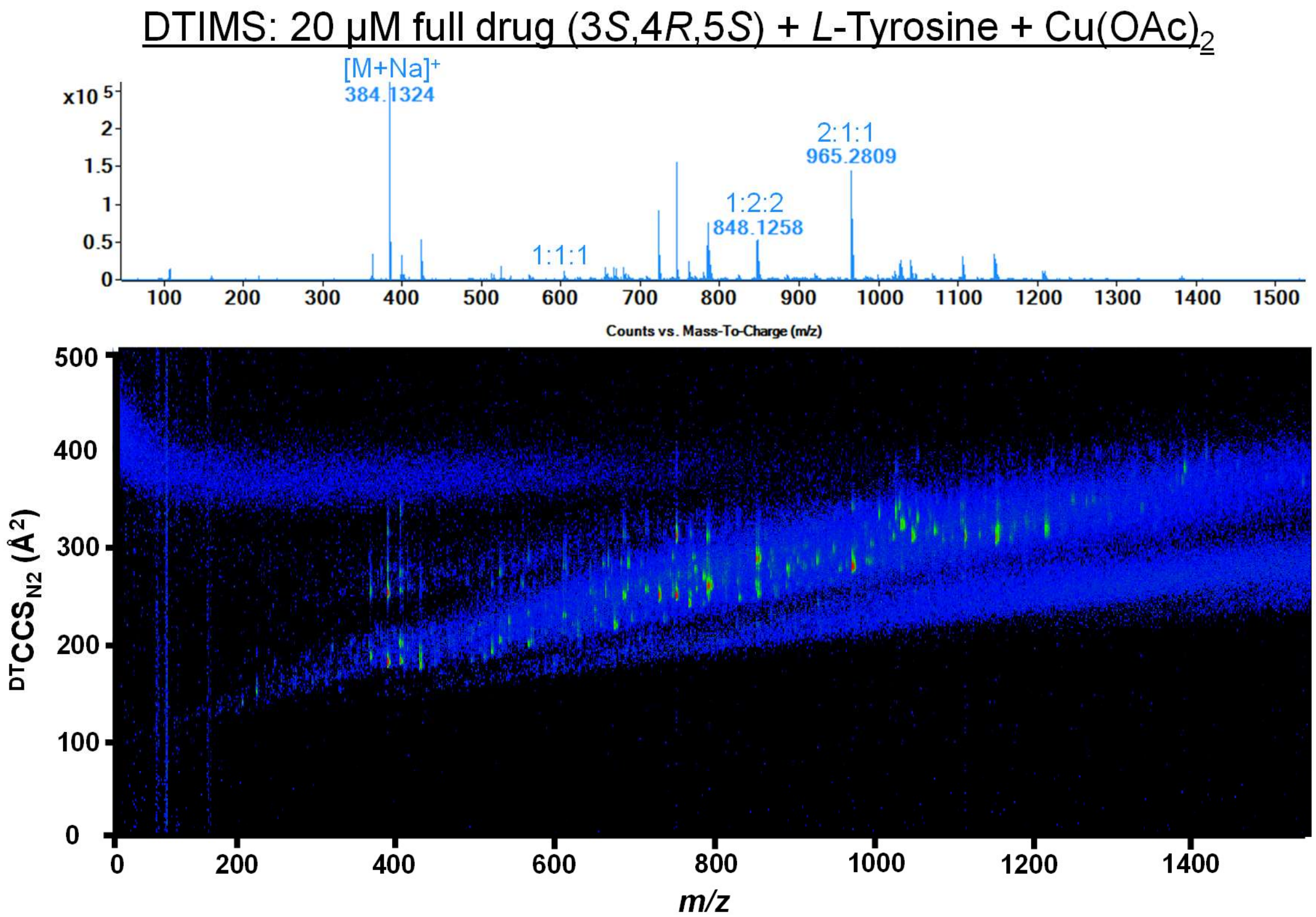

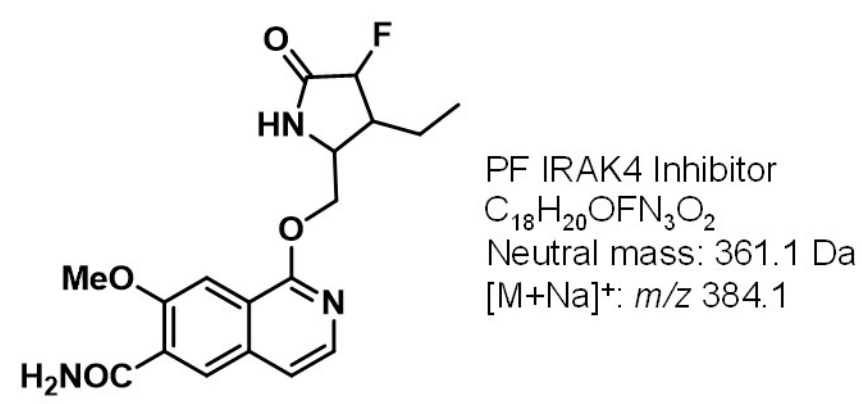

Figure S20. DTIMS-MS spectrum for the full drug with copper (II) acetate and *L*-tyrosine (all 20 μM in 1:1 methanol:water). Here, ratios listed above peaks indicate the stoichiometry of the corresponding drug:tyrosine:copper complex. For instance, a 1:2:2 annotation indicates a complex consisting of 1 analyte ligand, 2 tyrosine chiral reference ligands, and 2 copper ions (a binuclear complex).

Table S2. Measured $^{DT}CCS_{N2}$ values for observed ternary drug-copper-tyrosine complexes. Observations are organized by drug ligand. Complexes explicitly searched for but not observed are indicated with a "-".

(A) Copper-tyrosine complexes:

Full drug									
		$^{DT}CCS_{N2}$ (Å^2)							
**Complex**	***m/z***	**(3*S*,4*S*,5*S*)**	**(3*R*,4*R*,5*R*)**	**(3*R*,4*S*,5*S*)**	**(3*S*,4*R*,5*R*)**	**(3*S*,4*R*,5*S*)**	**(3*R*,4*S*,5*R*)**	**(3*R*,4*R*,5*S*)**	**(3*S*,4*S*,5*R*)**
[(M)(*L*-Tyr)(Cu^{II})-H]$^+$	604.1	227.00	232.80	229.42	230.73	228.25	233.08	230.44	232.39
[(M)$_2$(*L*-Tyr)(Cu^{II})-H]$^+$	965.3	290.63	288.22	279.51	286.34	285.82	288.16	277.97	286.41
[(M)(*L*-Tyr)$_2$(Cu^{II})-H]$^+$	785.2	-	-	-	-	-	-	-	-
[(M)(*L*-Tyr)(Cu^{II})+Na-2H]$^+$	626.1	-	-	-	-	-	-	-	-
[(M)(*L*-Tyr)$_2$(Cu^{II})$_2$-3H]$^+$	846.1	272.71	272.89	288.12	288.16	253.08	253.09	270.39	270.52
[(M)$_2$(*L*-Tyr)$_2$(Cu^{II})$_2$-3H]$^+$	1207.3	-	-	-	-	-	-	-	-
[(M)(*L*-Tyr)$_3$(Cu^{II})$_2$+Na-4H]$^+$	1049.1	-	-	-	-	-	-	-	-
[(M)$_3$(*L*-Tyr)$_2$(Cu^{II})$_3$-5H]$^+$	1629.3	-	-	-	-	-	-	-	-

γ-lactam scaffold									
		$^{DT}CCS_{N2}$ (Å^2)							
**Complex**	***m/z***	**(3*S*,4*S*,5*S*)**	**(3*R*,4*R*,5*R*)**	**(3*R*,4*S*,5*S*)**	**(3*S*,4*R*,5*R*)**	**(3*S*,4*R*,5*S*)**	**(3*R*,4*S*,5*R*)**	**(3*R*,4*R*,5*S*)**	**(3*S*,4*S*,5*R*)**
[(M)(*L*-Tyr)(Cu^{II})-H]$^+$	404.1	175.54	179.63	178.06	177.97	177.57	178.48	178.82	177.13
[(M)$_2$(*L*-Tyr)(Cu^{II})-H]$^+$	565.2	218.44	210.79	216.56	214.62	219.62	211.57	214.92	214.16
[(M)(*L*-Tyr)$_2$(Cu^{II})-H]$^+$	585.2	-	-	-	-	-	-	-	-
[(M)(*L*-Tyr)(Cu^{II})+Na-2H]$^+$	426.1	179.34	184.86	182.45	181.13	180.62	183.52	183.24	180.82
[(M)(*L*-Tyr)$_2$(Cu^{II})$_2$-3H]$^+$	646.1	225.57	225.73	219.19	225.36	225.31	221.01	220.45	224.17
[(M)$_2$(*L*-Tyr)$_2$(Cu^{II})$_2$-3H]$^+$	807.2	252.09	252.70	250.42	250.83	250.83	249.22	252.33	250.63
[(M)(*L*-Tyr)$_3$(Cu^{II})$_2$+Na-4H]$^+$	849.1	-	-	-	-	-	-	-	-
[(M)$_3$(*L*-Tyr)$_2$(Cu^{II})$_3$-5H]$^+$	1029.2	-	-	-	-	-	-	-	-

(B) Copper-histidine complexes:

γ-lactam scaffold									
		$^{DT}CCS_{N2}$ (Å^2)							
**Complex**	***m/z***	**(3*S*,4*S*,5*S*)**	**(3*R*,4*R*,5*R*)**	**(3*R*,4*S*,5*S*)**	**(3*S*,4*R*,5*R*)**	**(3*S*,4*R*,5*S*)**	**(3*R*,4*S*,5*R*)**	**(3*R*,4*R*,5*S*)**	**(3*S*,4*S*,5*R*)**
[(M)(*L*-Tyr)(Cu^{II})-H]$^+$	378.1	175.67	173.14	176.81	174.66	176.44	174.37	176.18	175.10
[(M)$_2$(*L*-Tyr)(Cu^{II})-H]$^+$	539.2	-	-	-	-	-	-	-	-
[(M)(*L*-Tyr)$_2$(Cu^{II})-H]$^+$	533.1	-	-	-	-	-	-	-	-
[(M)(*L*-Tyr)(Cu^{II})+Na-2H]$^+$	400.1	175.52	175.71	178.12	176.97	177.50	176.35	178.18	176.78
[(M)(*L*-Tyr)$_2$(Cu^{II})$_2$-3H]$^+$	594.1	202.56	208.17	206.36	207.02	203.71	207.70	207.11	206.67
[(M)$_2$(*L*-Tyr)$_2$(Cu^{II})$_2$-3H]$^+$	755.1	-	-	-	-	-	-	-	-
[(M)(*L*-Tyr)$_3$(Cu^{II})$_2$+Na-4H]$^+$	771.1	-	-	-	-	-	-	-	-
[(M)$_3$(*L*-Tyr)$_2$(Cu^{II})$_3$-5H]$^+$	977.1	-	-	-	-	-	-	-	-

Table S3. Interday reproducibility of the CCS values obtained in this work from the two ion mobility instrument platforms.

	Lactam (3*S*,4*R*,5*S*): $[(M)_2(L\text{-Tyr})(Cu^{II})\text{-H}]^+$	
	Interday CCS Measurements (Å^2)	
	**DTIMS**	**TWSLIM**
**R1**	219.62	217.50
**R2**	218.68	217.88
**R3**	218.64	217.33
**Avg.**	218.98	217.57
**RSD (%)**	0.21%	0.11%

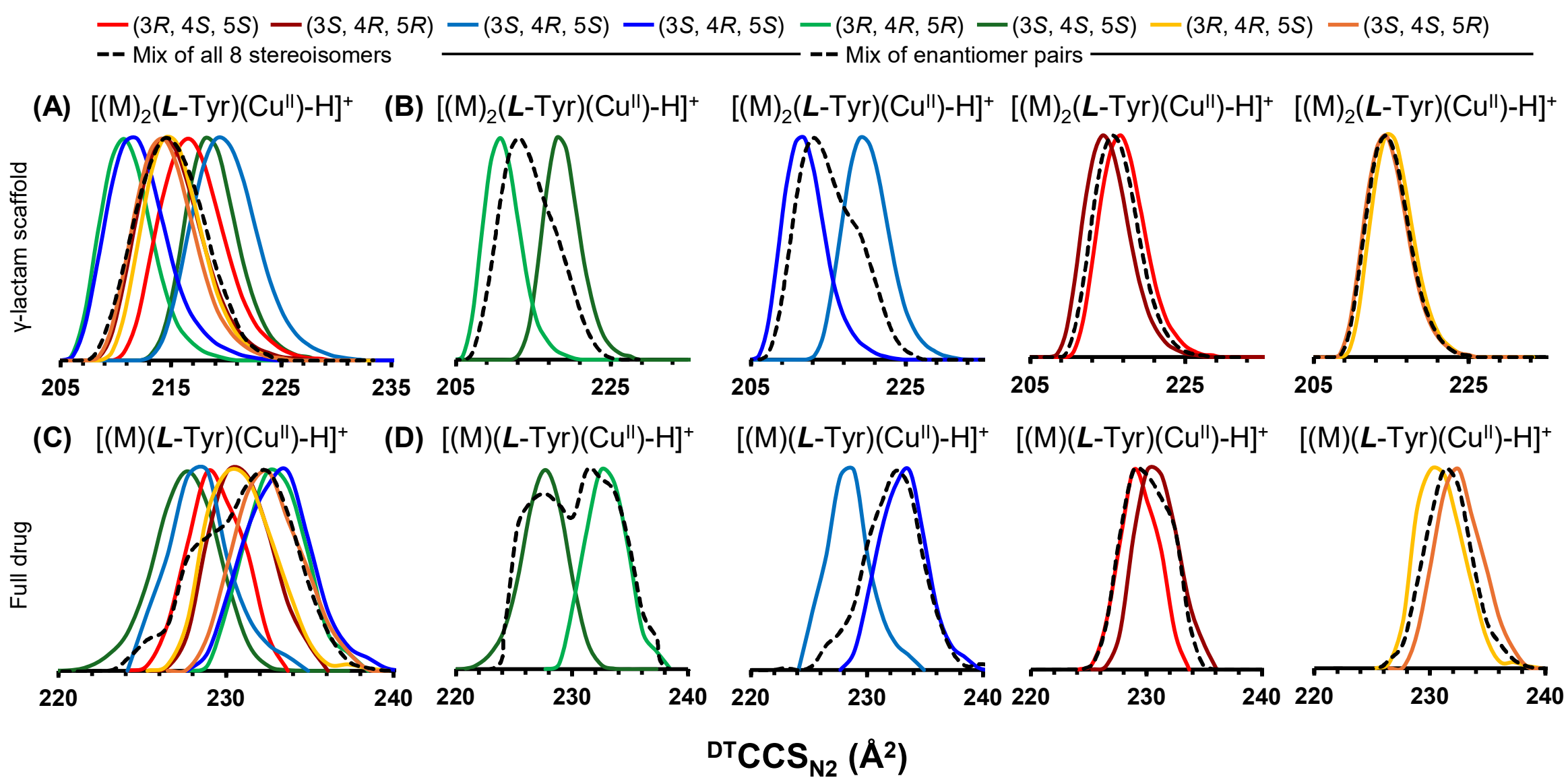

Figure S21. Differentiation achieved for copper-tyrosine complexes of interest using DTIMS-MS. **(A)** DTIMS spectra for enantiopure standards of the γ-lactam scaffold, as well as a mixture of all 8 stereoisomers, complexed as a dimer with copper-tyrosine in the form $[(M)_2(L\text{-Tyr})(Cu^{II})\text{-H}]^+$. **(B)** DTIMS differentiation of enantiomer pairs of the γ-lactam scaffold using $[(M)_2(L\text{-Tyr})(Cu^{II})\text{-H}]^+$. **(C)** DTIMS spectra for enantiopure standards of the full drug, as well as a mixture of all 8 stereoisomers, complexed as a monomer with copper-tyrosine in the form $[(M)(L\text{-Tyr})(Cu^{II})\text{-H}]^+$. **(D)** DTIMS differentiation of enantiomer pairs of the full drug using $[(M)(L\text{-Tyr})(Cu^{II})\text{-H}]^+$.

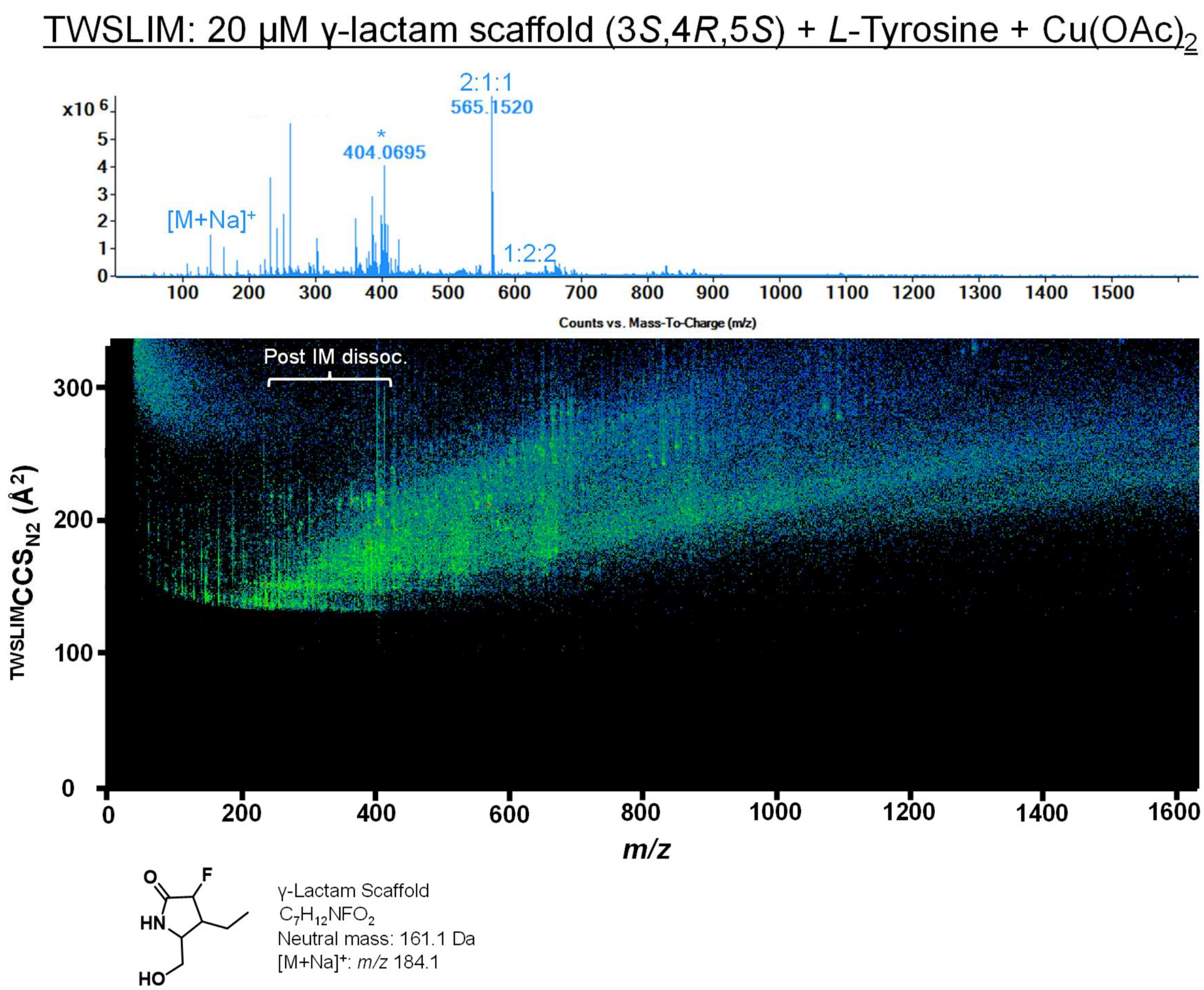

Figure S22. TWSLIM-MS spectrum for the γ-lactam scaffold with copper (II) acetate and *L*-tyrosine (all 20 µM in 1:1 methanol:water). Here, ratios listed above some peaks indicate the stoichiometry of the corresponding drug:tyrosine:copper complex. For instance, a 2:1:1 annotation indicates a complex consisting of 2 analyte ligands, 1 tyrosine chiral reference ligands, and 1 copper ions (a mononuclear dimer-incorporated complex). Here, the copper-tyrosine monomer (indicated with an asterisk) is only observed as a consequence of post-mobility dissociation.

TWSLIM: 20 µM full drug (3*S*,4*R*,5*S*) + *L*-Tyrosine + $Cu(OAc)_2$

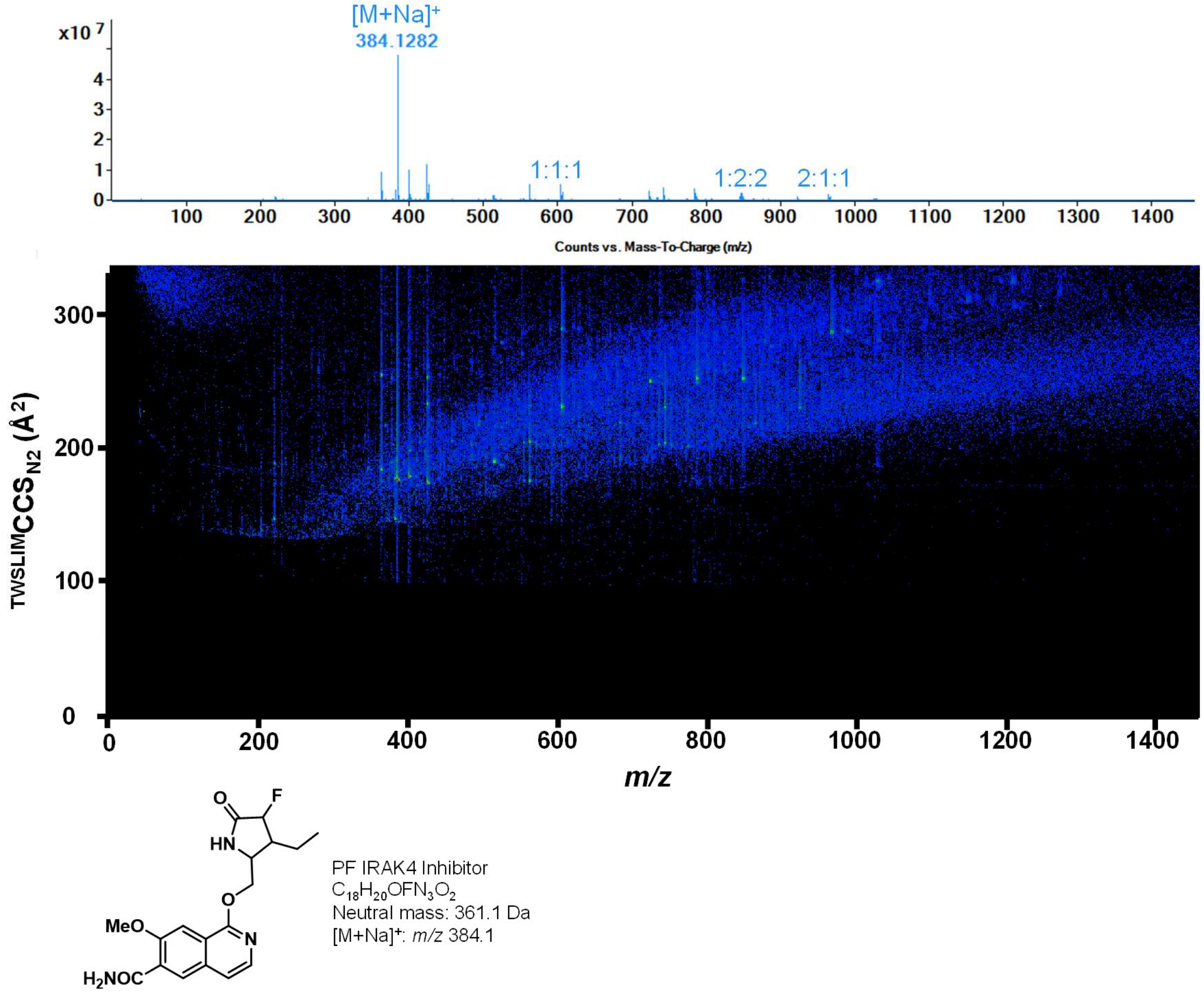

Figure S23. TWSLIM-MS spectrum for the full drug with copper (II) acetate and *L*-tyrosine (all 20 µM in 1:1 methanol:water). Here, ratios listed above peaks indicate the stoichiometry of the corresponding drug:tyrosine:copper complex. For instance, a 1:1:1 annotation indicates a complex consisting of 1 analyte ligand, 1 tyrosine chiral reference ligands, and 1 copper ions (a mononuclear ternary complex).

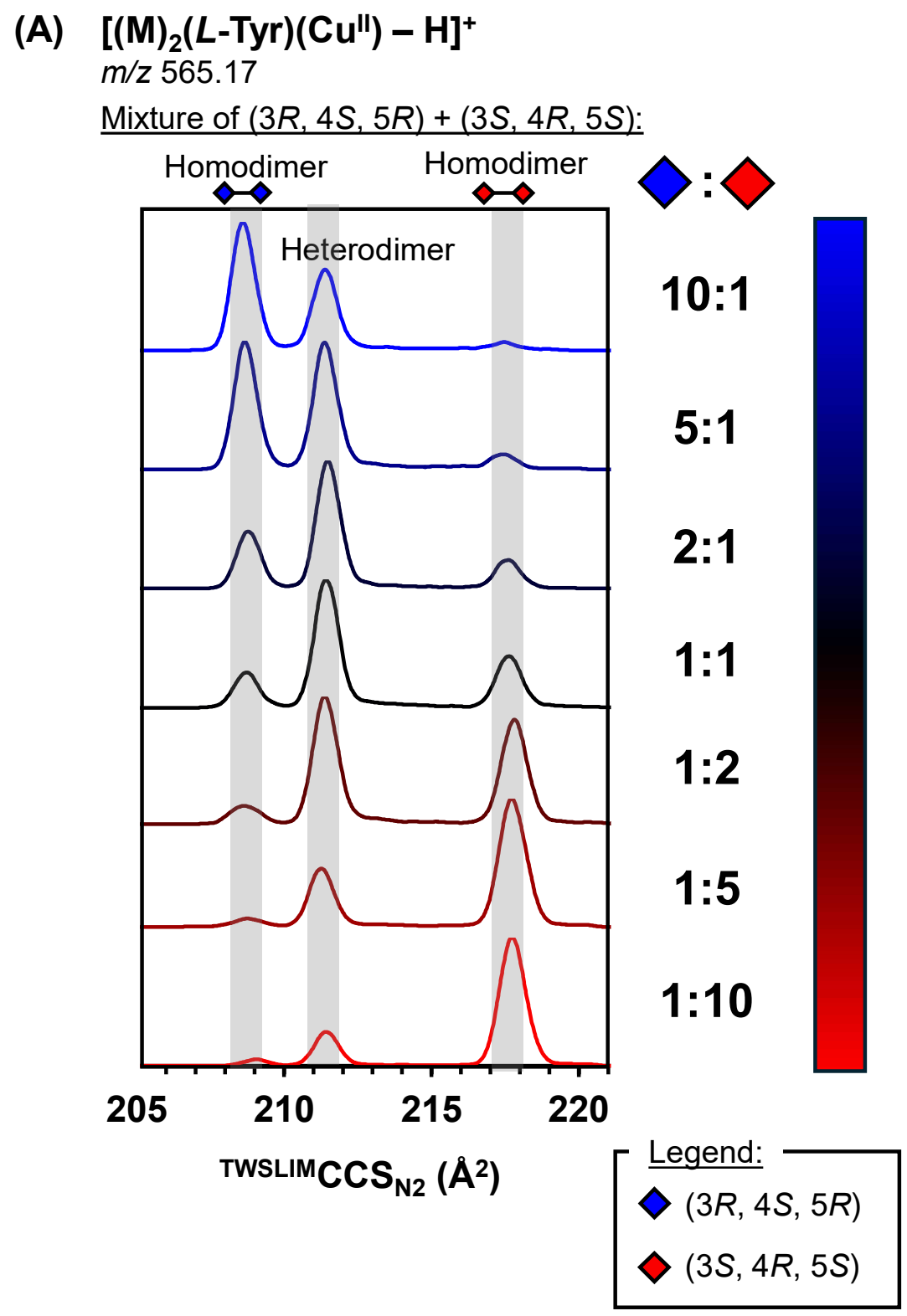

(B) Preliminary quantitation studies

$$\%\ Enantiomer = \frac{A_{homo} + \frac{1}{2}A_{hetero}}{A_{total}} \text{ x } 100\%$$

Mane, S.S.; Dearden, D.V.; Lee, K.W. *JASMS*, **2024** *35* (10), 2458-2464.

Sample	Measured					Predicted	
	3R, 4S, 5R	Heterodimer	*3S ,4R ,5S*	Total area	Hetero / 2	Percent 3R,4S,5R	Percent 3S,4R,5S
10:1	2224947.99	1468211.92	130773.69	3823933.6	734105.96	77%	23%
5:1	1464937.47	1498158.91	157518.88	3120615.3	749079.46	71%	29%
2:1	722472.04	1645085.26	372316.91	2739874.2	822542.63	56%	44%
1:1	444585.85	1476303.05	693945.78	2614834.7	738151.53	45%	55%
1:2	220838.86	1416000.13	1194282.47	2831121.5	708000.07	33%	67%
1:5	135521.81	1054145.63	2478179.66	3667847.1	527072.82	18%	82%
1:10	113233.61	810597.02	3393056.78	4316887.4	405298.51	12%	88%

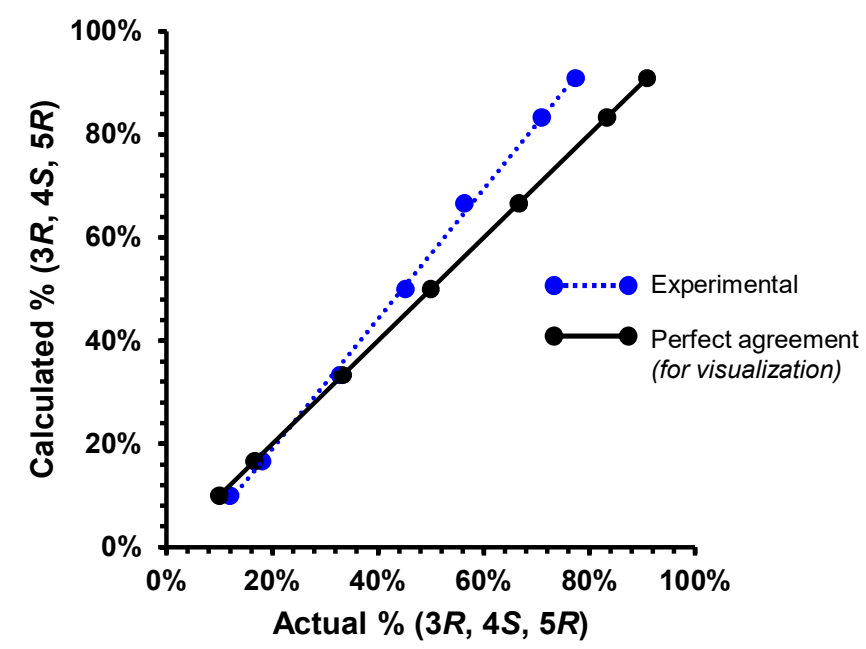

Figure S24. TWSLIM spectra for the γ-lactam scaffold complexed to a copper-tyrosine dimer of the form $[(M)_2(L\text{-Tyr})(Cu^{II})\text{-H}]^+$. Shown are profiles for a mixture of enantiomers (3*R*, 4*S*, 5*R*) and (3*S*, 4*R*, 5*S*) at varying ratios, including 10:1, 5:1, 2:1, 1:1, 1:2, 1:5, and 1:10. Preliminary quantitation using a protocol published by Kenneth Lee and coworkers in 2024 enabled preliminary quantitation of enantiomeric excess using these results.

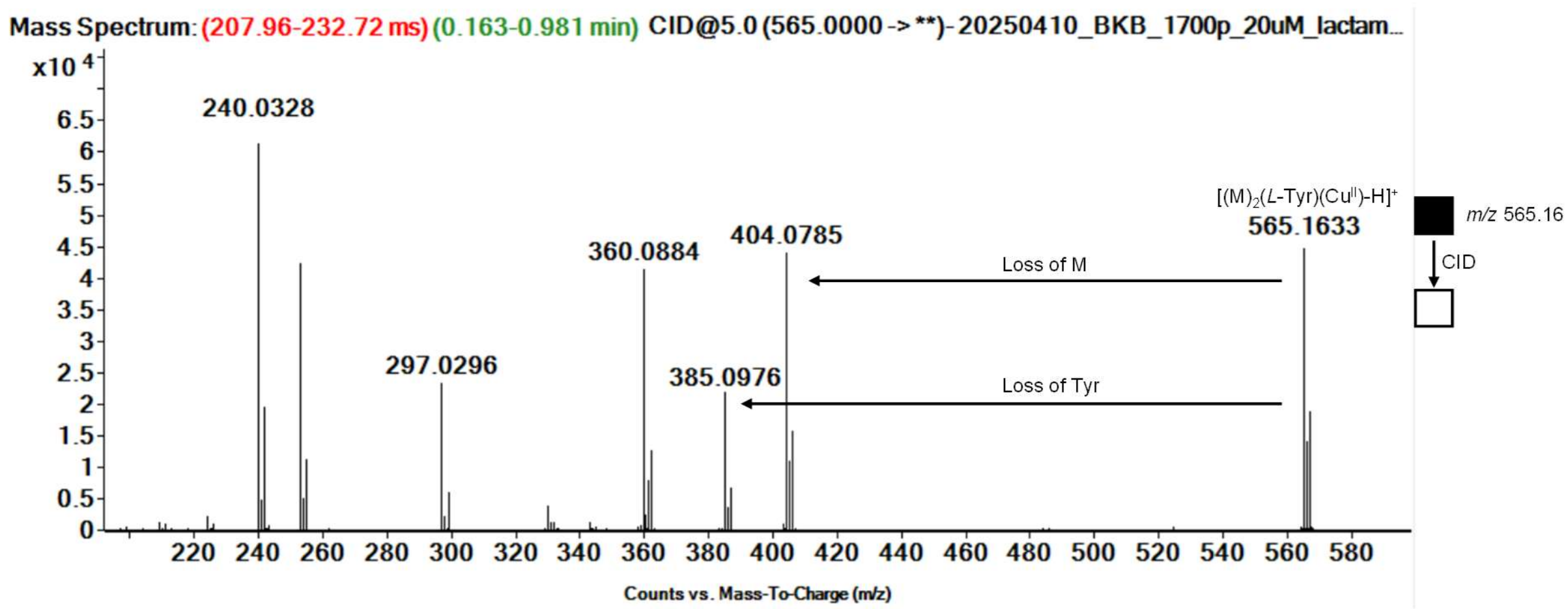

Figure S25. Collision-induced dissociation of the lactam's $[(M)_2(L\text{-Tyr})(Cu^{II})\text{-H}]^+$ results in two distinct fragmentation pathways, including the loss of an analyte ligand and the loss of a tyrosine reference ligand. This fragmentation is representative of all lactam complexes evaluated in this study.

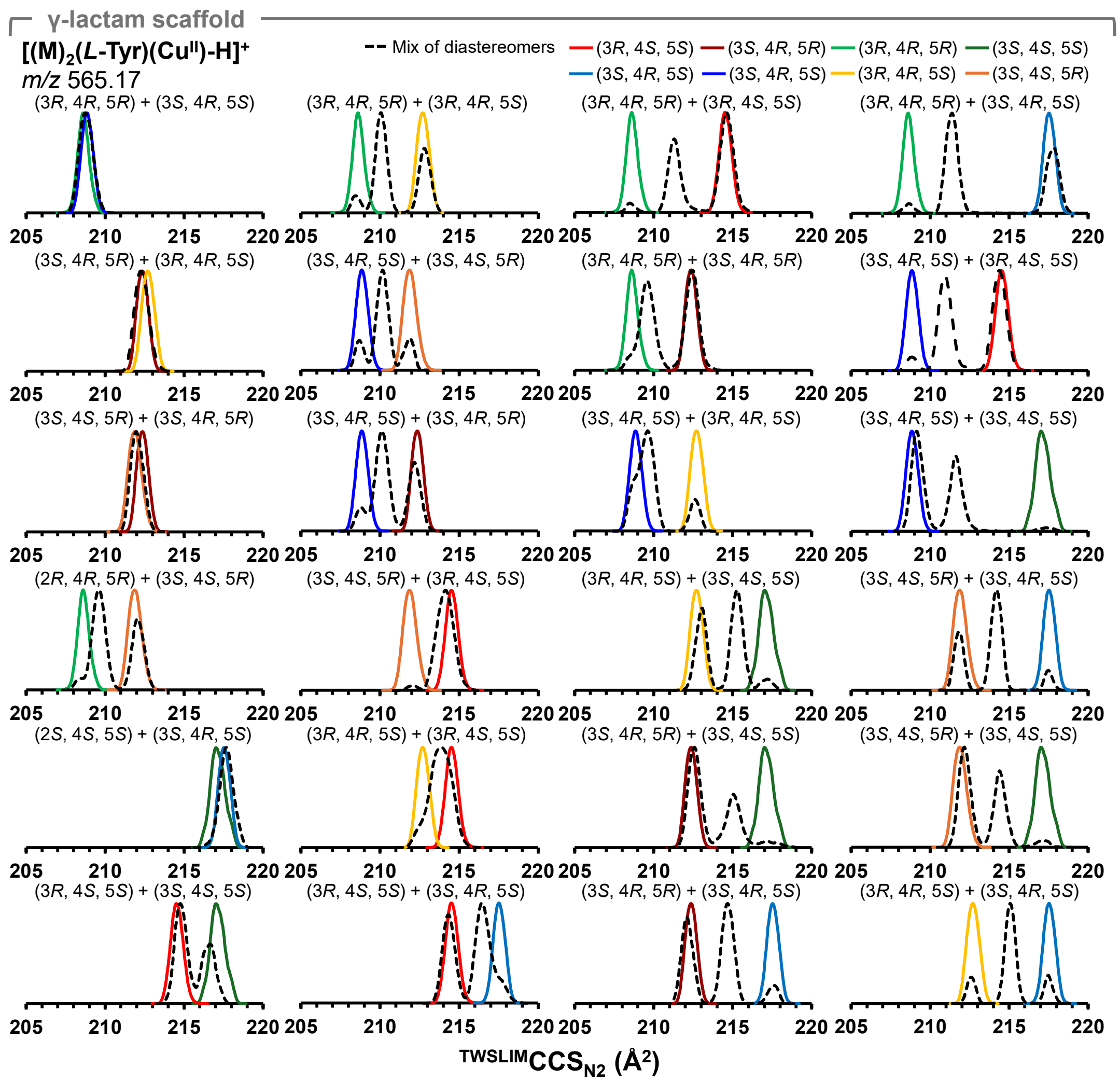

Figure S26. TWSLIM spectra for the γ-lactam scaffold complexed to a copper-tyrosine dimer of the form $[(M)_2(L\text{-Tyr})(Cu^{II})\text{-}H]^+$. Each of the 24 spectra is obtained from a different binary combination of diastereomers, enabling the elucidation of each possible heterodimer complex present in a mixture of all 8 stereoisomers.

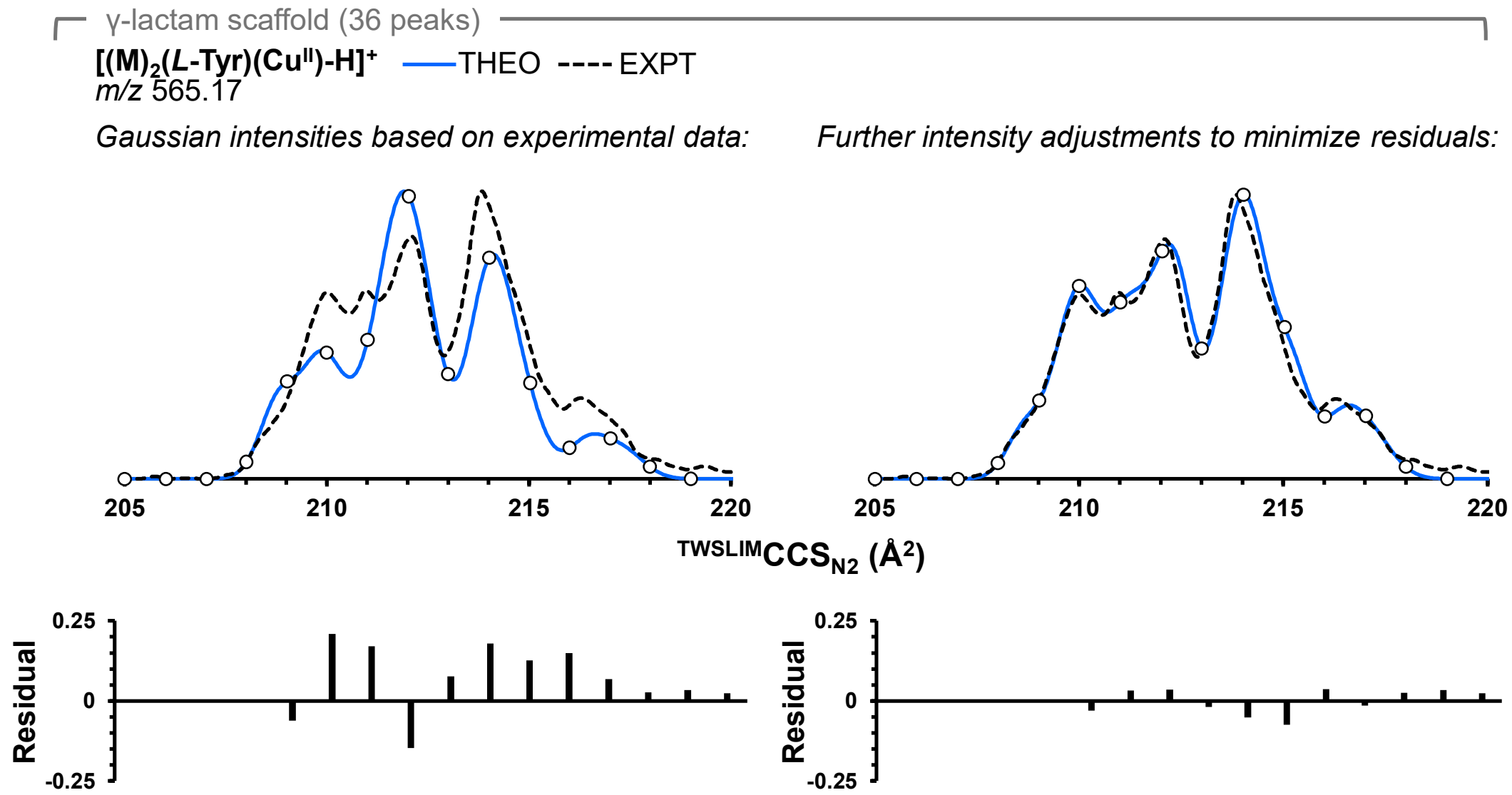

Figure S27. Example of fitting optimization to minimize residuals. Here, the predicted spectrum was constructed by first normalizing individual Gaussian fits to the intensities experimentally observed from the IM analysis of each binary mixture of diastereomers. Then, these intensities were further adjusted to minimize the residuals.

Table S4. Peak statistics (μ=centroid, σ=peak width, $R_P=\mu/(\sigma*2.3548)$ =single peak resolving power) obtained from fitting Gaussians to experimental data. Heterodimers were incorporated into the fits for the binary mixture data.

	γ-lactam scaffold				
	Sample Information		Peak Fitting		
	Peak #	Stereoisomers in Sample	μ	σ	R_P
Enantiopure	1	(3*S*, 4*S*, 5*S*)	225.39	0.45	213
	2	(3*R*, 4*R*, 5*R*)	231.44	0.49	201
	3	(3*R*, 4*S*, 5*S*)	227.43	0.45	215
	4	(3*S*, 4*R*, 5*R*)	229.30	0.43	227
	5	(3*S*, 4*R*, 5*S*)	226.54	0.45	214
	6	(3*R*, 4*S*, 5*R*)	231.36	0.47	209
	7	(3*R*, 4*R*, 5*S*)	228.99	0.45	216
	8	(3*S*, 4*S*, 5*R*)	230.72	0.45	218
		Average			214

	Full Drug				
	Sample Information		Peak Fitting		
	Peak #	Stereoisomers in Sample	μ	σ	R_P
Enantiopure	1	(3*S*, 4*S*, 5*S*)	217.02	0.470	196
	2	(3*R*, 4*R*, 5*R*)	208.60	0.360	246
	3	(3*R*, 4*S*, 5*S*)	214.52	0.380	240
	4	(3*S*, 4*R*, 5*R*)	212.37	0.390	231
	5	(3*S*, 4*R*, 5*S*)	217.53	0.375	246
	6	(3*R*, 4*S*, 5*R*)	208.85	0.373	238
	7	(3*R*, 4*R*, 5*S*)	212.70	0.395	229
	8	(3*S*, 4*S*, 5*R*)	211.86	0.410	219
Ena. Mixes	9	(3*S*, 4*S*, 5*S*) + (3*R*, 4*R*, 5*R*)	211.48	0.400	225
	10	(3*R*, 4*S*, 5*S*) + (3*S*, 4*R*, 5*R*)	213.81	0.393	231
	11	(3*S*, 4*R*, 5*S*) + (3*R*, 4*S*, 5*R*)	211.60	0.390	230
	12	(3*R*, 4*R*, 5*S*) + (3*S*, 4*S*, 5*R*)	212.03	0.393	229
Diastereomer mixes	13	(3*S*, 4*S*, 5*S*) + (3*R*, 4*S*, 5*S*)	216.55	0.393	234
	14	(3*S*, 4*S*, 5*S*) + (3*S*, 4*R*, 5*R*)	215.02	0.430	212
	15	(3*S*, 4*S*, 5*S*) + (3*S*, 4*R*, 5*S*)	217.28	0.393	235
	16	(3*S*, 4*S*, 5*S*) + (3*R*, 4*S*, 5*R*)	211.64	0.390	230
	17	(3*S*, 4*S*, 5*S*) + (3*R*, 4*R*, 5*S*)	215.28	0.380	241
	18	(3*S*, 4*S*, 5*S*) + (3*S*, 4*S*, 5*R*)	214.39	0.380	240
	19	(3*R*, 4*R*, 5*R*) + (3*R*, 4*S*, 5*S*)	211.30	0.380	236
	20	(3*R*, 4*R*, 5*R*) + (3*S*, 4*R*, 5*R*)	209.57	0.440	202
	21	(3*R*, 4*R*, 5*R*) + (3*S*, 4*R*, 5*S*)	211.39	0.390	230
	22	(3*R*, 4*R*, 5*R*) + (3*R*, 4*S*, 5*R*)	208.73	0.393	225
	23	(3*R*, 4*R*, 5*R*) + (3*R*, 4*R*, 5*S*)	210.08	0.360	248
	24	(3*R*, 4*R*, 5*R*) + (3*S*, 4*S*, 5*R*)	209.61	0.420	212
	25	(3*R*, 4*S*, 5*S*) + (3*S*, 4*R*, 5*S*)	216.46	0.420	219
	26	(3*R*, 4*S*, 5*S*) + (3*R*, 4*S*, 5*R*)	210.92	0.410	218
	27	(3*R*, 4*S*, 5*S*) + (3*R*, 4*R*, 5*S*)	213.84	0.393	231
	28	(3*R*, 4*S*, 5*S*) + (3*S*, 4*S*, 5*R*)	214.14	0.393	231
	29	(3*S*, 4*R*, 5*R*) + (3*S*, 4*R*, 5*S*)	214.69	0.390	234
	30	(3*S*, 4*R*, 5*R*) + (3*R*, 4*S*, 5*R*)	210.12	0.410	218
	31	(3*S*, 4*R*, 5*R*) + (3*R*, 4*R*, 5*S*)	212.54	0.393	229
	32	(3*S*, 4*R*, 5*R*) + (3*S*, 4*S*, 5*R*)	211.98	0.393	229
	33	(3*S*, 4*R*, 5*S*) + (3*R*, 4*R*, 5*S*)	215.07	0.340	269
	34	(3*S*, 4*R*, 5*S*) + (3*S*, 4*S*, 5*R*)	214.18	0.350	260
	35	(3*R*, 4*S*, 5*R*) + (3*R*, 4*R*, 5*S*)	209.61	0.430	207
	36	(3*R*, 4*S*, 5*R*) + (3*S*, 4*S*, 5*R*)	210.16	0.360	248
		Average			230

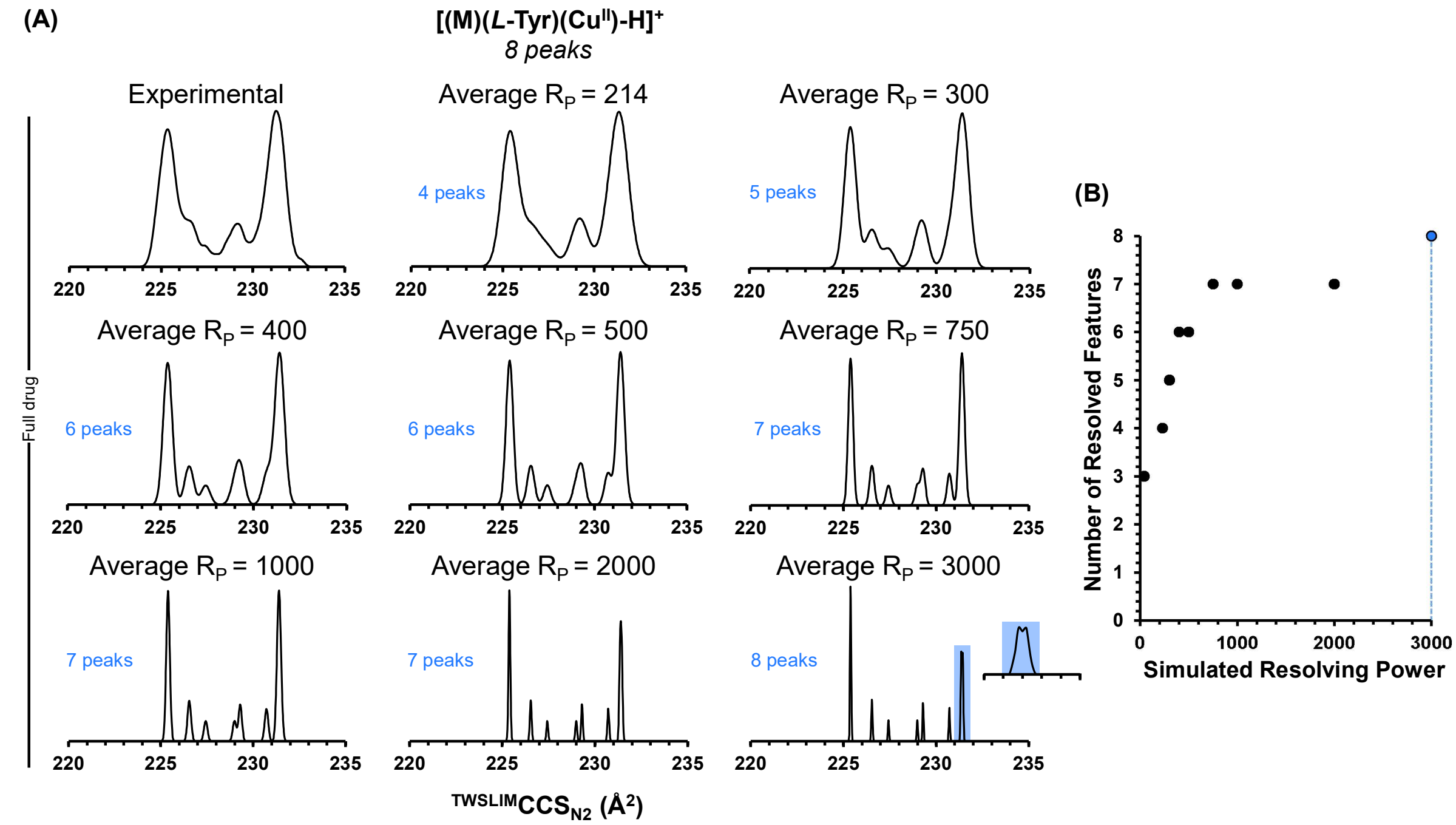

Figure S28. (A) Simulated IM spectra of a mixture of all stereoisomers of Zimlovisertib containing 8-peaks with increasingly higher IM resolving powers. Here, a scaling factor was applied to σ values until target average R_P values were achieved. Peak fitting results indicated that 5 peaks are resolvable at an R_P of 300, and 7 peaks are resolvable by an R_P of 750. However, elucidation of (3*R*,4*R*,5*R*) and (3*R*,4*S*,5*R*) stereoisomers ($\Delta CCS_{\%}$ = 0.035%) is not possible until an R_P of nearly 3000. **(B)** The number of resolved features plotted against the simulated resolving power. The dashed line in blue indicates the resolving power at which all 8 features are resolvable, 3000.

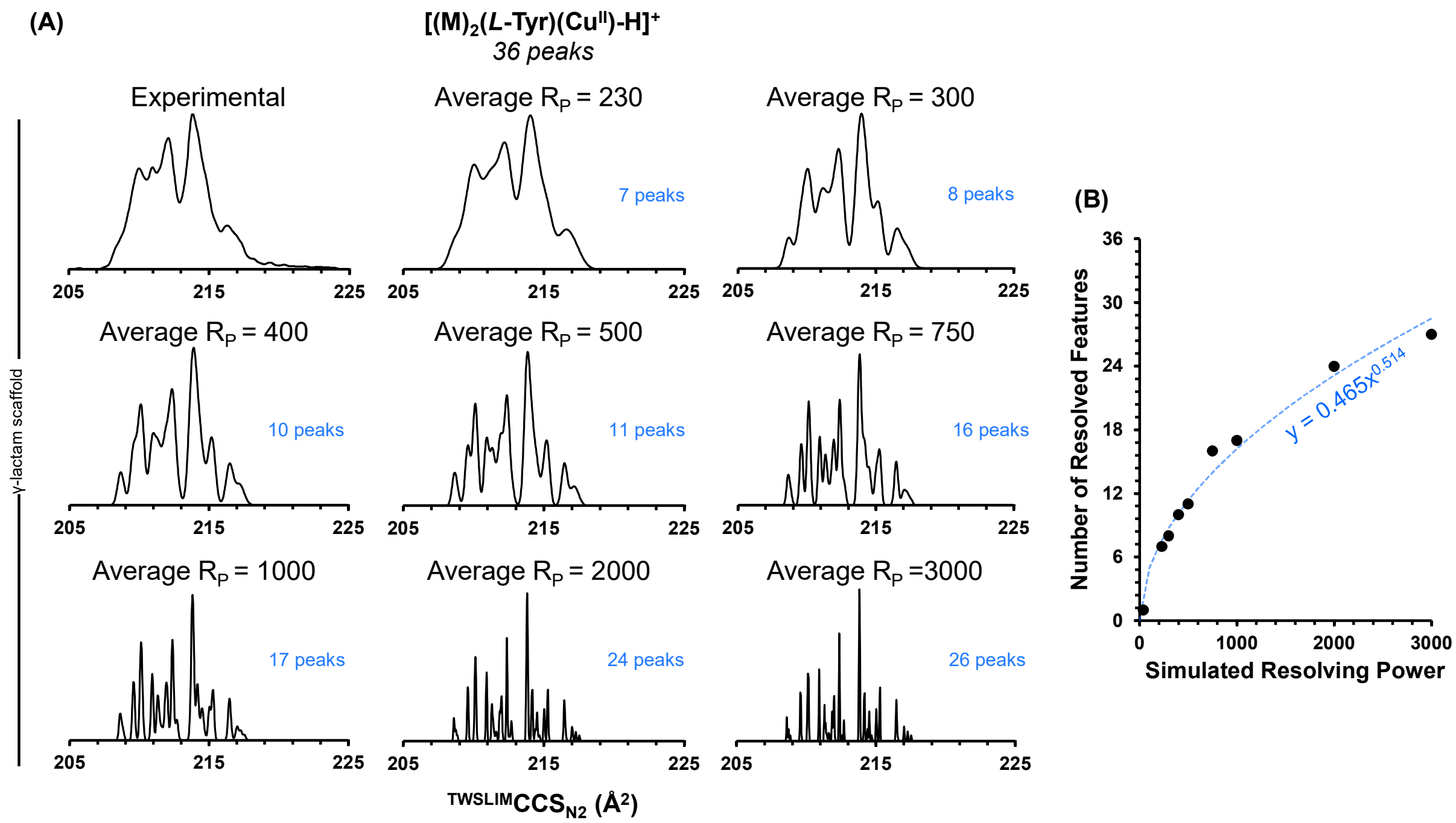

Figure S29. (A) Simulated IM spectra of the γ-lactam scaffold with increasingly higher IM resolving powers towards the differentiation of a mixture spectrum containing 36 features. While direct resolution of all 36 features is not possible due to two heterodimers exhibiting the same CCS (peaks #24 and #35 in Table S3 both have a $^{TWSLIM}CCS_{N2}$ of 209.61 Å^2), one can see in **(B)** that the resolving power required to resolve 35 out of the 36 peaks can be extrapolated via power fit to be approximately 4500.
